# Optimized SnO_2_ Thin Films: Correlating Solution Chemistry and Deposition Conditions with Optoelectronic Properties

**DOI:** 10.1021/acsami.6c03389

**Published:** 2026-03-26

**Authors:** Iqra Ramzan, Matthew O. Blunt, Ivan P. Parkin, Claire J. Carmalt

**Affiliations:** Materials Chemistry Centre, Department of Chemistry, 4919 University College London, 20 Gordon Street, London WC1H 0AJ, U.K.

**Keywords:** transparent conducting oxides (TCOs), optoelectronic properties, tin oxide (SnO_2_) thin films, aerosol-assisted chemical vapor deposition (AACVD), transparent electrodes, photovoltaic applications, thin-film deposition, solvent effects

## Abstract

The ability to deposit high-quality SnO_2_ thin films using simple, scalable techniques such as aerosol-assisted chemical vapor deposition (AACVD) has increased its potential for optoelectronic applications, including photovoltaics, displays, and smart windows. However, optimizing the structural and electronic properties of SnO_2_ requires a deeper understanding of how deposition parameters influence film growth. To the best of our knowledge, the optimization of SnO_2_ films using SnCl_4_ as a precursor via AACVD has been largely overlooked in the literature. This study addresses that gap by selectively investigating the effects of solvent composition, deposition temperature, and misting time on the microstructure, optical transparency, and electrical performance of the SnO_2_ films. Residual chlorine in the films was detected and quantified. The results demonstrate that careful control of solution chemistry and deposition conditions enabled the fabrication of SnO_2_ films with high transparency and low resistivity, comparable to those of doped systems. Importantly, these insights can also pave the way for further optimization of commercially available fluorine-doped tin oxide (FTO) films, which are widely deposited via CVD, extending the industrial relevance of this work.

## Graphical abstract

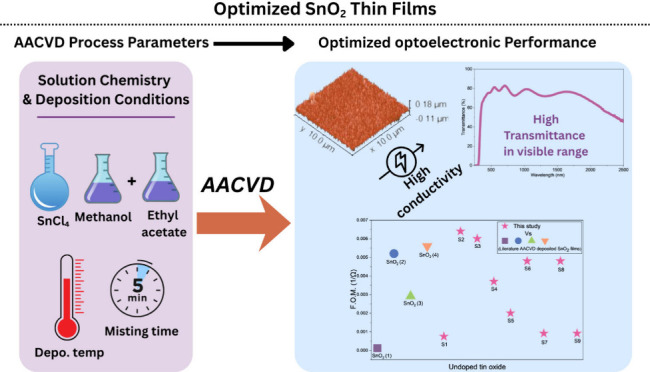

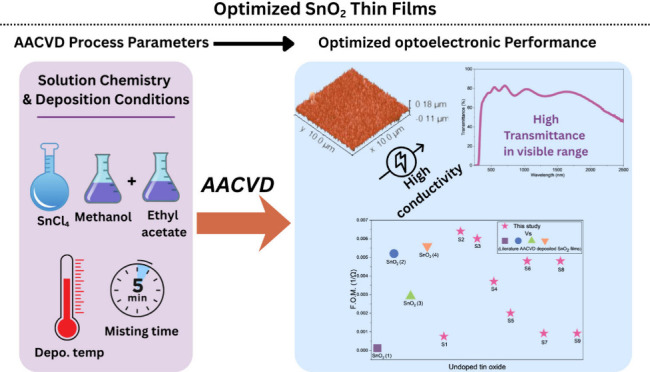

## Introduction

Transparent conducting oxide (TCO) is an essential component in optoelectronic devices such as photovoltaics, flat-panel displays, and smart windows, owing to their rare combination of high optical transparency and electrical conductivity.^1^ Developing efficient and sustainable TCO materials is of considerable commercial importance, given their widespread applications. To qualify as a TCO, a material must possess high optical transparency (>70%) in the visible region (380–750 nm), low electrical resistivity (<10^–3^ Ω cm), high electron mobility (>10 cm^2^ V^–1^ s^–1^), and a reasonably high carrier concentration (<10^22^ cm^–3^).^1^ The high conductivity of these materials is governed by both the density of free carriers and their mobility.^1^

Indium tin oxide (ITO) has been widely used as a TCO material for several decades. However, the limited supply of indium, along with the continuous demand for performance improvements, has accelerated research into finding alternative TCO materials.^1,2^ Tin oxide (SnO_2_), which is abundant, inexpensive, and nontoxic, has gained immense attention. SnO_2_ is a wide band gap semiconductor, reported as intrinsically n-type, and possesses properties that can be tuned through selective doping.^3^ SnO_2_ films are particularly preferred because they are resistant to chemical attack and thermal degradation and can retain strong mechanical durability.^4,5^ These properties make them attractive for thin-film applications in commercial products and a cost-effective alternative to the more expensive indium–tin oxide (ITO) films.^4,5^

The intrinsic n-type conductivity of SnO_2_ is associated with the presence of defect states such as oxygen vacancies or Sn-interstitials. This n-type conductivity can be improved by adding extrinsic dopants such as fluorine and antimony, which have been reported widely as potential dopants to enhance the conductivity of SnO_2_.^6,7^ However, excessive doping is not desirable as it can negatively affect conductivity by degrading the film structure, which leads to a reduction in the mobility of free charge carriers.^8,9^ Another challenge with doped SnO_2_ films is the uneven distribution of dopants across the substrate, which leads to nonuniform conductivity within the film.^10^

This study focuses on the deposition and optimization of SnO_2_ films via aerosol-assisted chemical vapor deposition (AACVD) using SnCl_4_ as the tin precursor. SnCl_4_ has previously been used in a variety of deposition techniques to deposit SnO_2_ films. For example, spray pyrolysis using SnCl_4_ achieved resistivity values of 8 × 10^–4^ Ω cm with ethanol as the solvent,^10^ 1 × 10^–2^ Ω cm with methanol,^7^ and 4.5 × 10^–3^ Ω cm with propan-2-ol.^6^ In atmospheric-pressure CVD (APCVD), Davazoglou^11^ and Koutsogianni and Tsamakis^12^ also reported the use of SnCl_4_ as a precursor, obtaining minimum resistivities of 1.3 × 10^–3^ and 1.6 × 10^–3^ Ω cm, respectively. Similarly, atomic layer deposition (ALD) using SnCl_4_ yielded a minimum resistivity of 8.0 × 10^–2^ Ω cm.^13^ To the best of our knowledge, no systematic study has been reported on the deposition and optimization of SnO_2_ films using SnCl_4_ as a tin precursor in AACVD, considering parameters such as solvent type or solvent mixtures, deposition temperature, and misting time. The only related report available describes the use of SnCl_4_·5H_2_O as a precursor via AACVD, which produced films with a resistivity of 3 × 10^2^ Ω cm.^14^

The solution chemistry and deposition parameters were optimized to tune the optical and electrical properties of the SnO_2_ thin films. The resulting films were systematically characterized for their crystal structure, chemical composition, morphology, optical behavior, and electrical performance using X-ray diffraction (XRD), X-ray photoelectron spectroscopy (XPS), scanning electron microscopy (SEM), UV–vis spectroscopy, and Hall effect measurements, as described in detail in the experimental section.

Various techniques have been employed for depositing TCO thin films, including photochemical methods, sol–gel processing, CVD, spin coating, dip coating, and spray pyrolysis. Among these, AACVD stands out as a superior technique due to its simplicity, scalability, and cost-effectiveness. AACVD not only eliminates the need for highly volatile precursors but also enables the deposition of uniform, adherent films with excellent control over dopant incorporation. In addition, AACVD offers high growth rates and the ability to produce uniform thin films, making it a highly scalable and efficient method for industrial applications.

## Experimental Details

All chemicals were used as received without further purification: tin­(IV) chloride (SnCl_4_; 99.995%, Sigma-Aldrich), methanol (MeOH; 99.9% Fischer), ethyl acetate (EA; 99.8%, Fischer Scientific), and acetonitrile (MeCN; 99.8%, Fischer Scientific). Compressed air was used as supplied by BOC. The glass substrate was 3.2 mm thick, plain float glass with a 50 nm thick SiO_2_ barrier layer, supplied by Pilkington NSG.

### Synthesis

Depositions were carried out in a flat-bed, cold-walled tubular reactor, as shown in Figure 1. The graphite block containing a Whatman cartridge heater monitored by a Pt–Rh thermocouple was placed at the lower half of the reactor to control substrate temperature. Depositions were conducted on Pilkington silica-coated barrier glass (50 nm SiO_2_ coating on one side of float glass) to prevent undesirable ion leaching from the glass into the thin film. The substrates (15 cm × 4 cm) were cleaned using soapy water and isopropanol, followed by drying in a 70 °C oven. To promote the laminar flow of the aerosol and enhance thin-film quality, a second piece of barrier glass was suspended 8 mm above the substrate, with the silica-coated side facing downward. The precursor solution consisting of 0.05 mL of SnCl_4_ was dissolved in 14 mL of the chosen solvent(s) in a glass bubbler. An aerosol was generated by using a piezoelectric device and delivered into the reactor by using a carrier gas flow (compressed air) at a rate of 1 L/min. The substrate was heated to the desired temperature prior to deposition and then cooled to room temperature after deposition.

**Figure 1:**
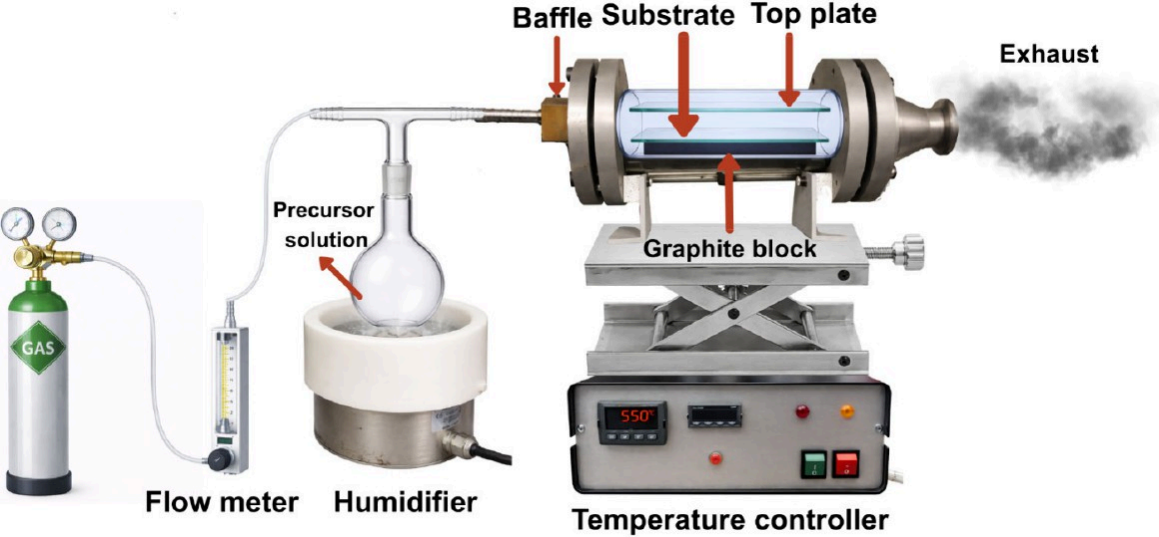
Schematic representation of the AACVD deposition setup.

### Analysis

Grazing-incident X-ray diffraction (GIXRD) measurements were conducted by using a Panalytical Empyrean diffractometer. The X-rays were generated using a Cu Kα source with a wavelength of 1.5406 Å, operating at a voltage of 40 kV, and an emission current of 40 mA. An incident beam angle of 1° was maintained during the measurements, and diffraction patterns were collected over a range of 10° to 70° (with 0.05° steps at 0.5 s/step). Peak positions were identified by comparison to standard data from the Inorganic Crystal Structure Database (ICSD). All the recorded patterns were analyzed to assess crystallinity and preferred growth orientation and were plotted using OriginPro software. The diffraction peaks were analyzed by using a Gauss peak profile. The peak positions (2θ) and full width at half-maximum (fwhm) values were extracted from the fitted profiles. Lattice parameters *a* and *c* were calculated from multiple diffraction peaks using Bragg’s law and the corresponding tetragonal unit-cell calculations. The uncertainties were estimated from the standard deviation of values obtained from different reflections, yielding an estimated uncertainty of ±0.001 Å. Unit cell volumes were calculated using *V* = *a* ^2^ *c*, and the associated uncertainties (±0.04 Å^3^) were determined by standard error propagation from the lattice parameters.

The average crystallite size was estimated using the Scherrer equation using the fwhm values obtained from Gauss-fitted peaks. Considering uncertainties in peak width determination, the crystallite sizes carry an estimated uncertainty of approximately ±10%.

X-ray photoelectron spectroscopy (XPS) analysis of the films was conducted by using a Thermo Scientific spectrophotometer equipped with a monochromatic Al Kα radiation source. High-resolution surface scans were conducted for the Sn 3d, Cl 2p, O 1s, and C 1s regions to determine the chemical composition using a pass energy of 40 eV and a spot size of 400 μm. The peaks were modeled using Avantage Thermo Fisher scientific software and plotted using OriginPro. Binding energies of the peaks were calibrated relative to the adventitious carbon peak at 284.8 eV to compensate for charging effects. The core-level spectra were modeled using a mixed Gaussian–Lorentzian peak shape, and the fitting was performed using the Powell optimization algorithm. A Smart background subtraction was performed before fitting. The uncertainties in the XPS atomic percentages were estimated by repeating the peak fitting for the spectra collected from three different areas on each sample.

All room temperature UV–vis transmittance and reflectance spectra were recorded by using a Shimadzu 3600i plus spectrometer over a wavelength range of 200–2600 nm. The band gap of films was calculated from the recorded spectra in OriginPro using the Tauc plot method.

The morphologies of the films were analyzed using scanning electron microscopy (SEM) in a top-down configuration, with accelerating voltages ranging from 5 to 10 keV, on a JEOL JSM-7600 field emission (FE) microscope. The samples were carbon coated to improve the SEM imaging and EDS analysis accuracy. Energy dispersive X-ray spectroscopy (EDS) was used to quantify the samples, specifically to determine the oxygen-to-metal ratio and the amount of dopant (atom %) present in the films.

AFM measurements were performed using a Bruker Nexus system operated in the PeakForce Tapping mode. For each sample, five independent 10 μm × 10 μm scans were collected at different surface locations spaced ∼3 mm apart to ensure statistical reliability. Images were acquired at a scan speed of 1 line s^–1^ with a resolution of 512 × 512 pixels and a peak force set point of 5.94 nN. Force Modulation AFM probes with aluminum reflective coating (Budget Sensors, Multi75Al-G; tip radius ≈ 10 nm, resonant frequency 75 kHz, force constant 3 N m^–1^) were used. A second-order plane flatten was applied to all images prior to analysis, and the root-mean-square (RMS) roughness was extracted using the same procedure for each data set to ensure consistency. For each sample, the reported RMS roughness corresponds to the mean of the five independent scans and the error is given as the standard error of the mean. The WSxM 5.0 software was used to process images and set the scale values.^15^

The film thicknesses were determined by using the Bruker Dektak XT instrument, operating at room temperature. Film thickness was measured on the 1 × 1 cm region of each sample, and electrical properties were measured on adjacent 1 × 1 cm regions to ensure accurate and representative electrical properties data. From each 15 cm substrate, the first 2 cm section was used for thickness and electrical measurements, and the 3 to 6 cm region was used for optical measurements to keep the data consistent.

A four-point probe was used to conduct Hall effect measurements to determine the electronic properties of the films. These measurements were performed on an Ecopia HMS-3000 setup configured in the Van der Pauw configuration to measure parameters, including resistivity, mobility, and carrier concentration. Samples measuring 1 cm^2^ were used for the measurements under an input current of 1 mA and a calibrated magnetic field of 0.58 T.

Three films were deposited per condition to ensure the reproducibility and reliability of the results. The error bars presented in the tables reflect the standard deviations calculated from these three repeats.

Finally, static water contact angle measurements were performed using a Krüss DSAE droplet shape analyzer by the sessile drop method. Ultrapure water droplets with a volume of 5 μL were used under ambient conditions. Contact angles were determined from the captured droplet images using the instrument’s image analysis software.

## Results and Discussion

SnO_2_ films were deposited by AACVD using different solvents and solvent mixtures and tested in varying ratios and deposition temperatures to identify the best deposition conditions. The deposition parameters of all the films are summarized in Table 1. Samples **S1**–**S5** were deposited at 590 °C using ethyl acetate, methanol (MeOH), or their mixtures in varying ratios. **S6** was deposited with an ethyl acetate and acetonitrile (MeCN) mixture. The influence of the deposition temperature was studied by depositing films from the same ethyl acetate–MeOH (11:3) mixture at 590 °C (**S2**), 550 °C (**S8**), and 500 °C (**S9**). The effect of misting duration was also investigated using the same ethyl acetate–MeOH (11:3) solution and varying the deposition time for **S2** and **S7**. Additional depositions performed at 400 °C did not produce continuous SnO_2_ films, and the maximum temperature was limited to 590 °C due to the use of glass substrates. Therefore, the study focused on the temperature window of 500–590 °C.

**Table 1:** Deposition Parameters, Crystallite Size, Lattice Parameters, Unit Cell Volume, Atomic % of Tin Oxide, Tin Hydroxide, Chlorine Content, and O/Sn Ratio of SnO_2_ Thin Films Prepared by AACVD

sample	solvent composition	deposition temp (°C)	misting time (min)	crystallite diameter (nm)	*a* (±0.001 Å)	*c* (±0.001 Å)	unit cell volume (±0.04 Å^3^)	tin oxide (rel. at %) XPS	tin hydroxide (rel. at %) XPS	O/Sn (from XPS data)	Cl (at % from XPS data)
**S1**	ethyl acetate 14 mL	590	05	12.7 ± 1.3	4.739	3.181	71.47	63.9	36.1	2.00 ± 0.14	9.50 ± 0.67
**S2**	ethyl acetate 11 mL + methanol 3 mL	590	05	25.5 ± 2.6	4.741	3.180	71.48	76.1	23.9	1.40 ± 0.10	0.5 ± 0.04
**S3**	ethyl acetate 7 mL + methanol 7 mL	590	05	28.1 ± 2.8	4.741	3.190	71.72	75.0	25.0	1.40 ± 0.10	0.5 ± 0.03
**S4**	ethyl acetate 3 mL + methanol 11 mL	590	05	23.7 ± 2.4	4.737	3.181	71.41	69.9	30.1	1.30 ± 0.09	2.1 ± 0.15
**S5**	methanol 14 mL	590	05	19.7 ± 2.0	4.748	3.190	71.92	71.8	28.2	2.10 ± 0.15	0.8 ± 0.06
**S6**	ethyl acetate 11 mL + acetonitrile 3 mL	590	05	31.7 ± 3.2	4.735	3.187	71.48	72.2	27.8	1.40 ± 0.10	1.3 ± 0.09
**S7**	ethyl acetate 11 mL + methanol 3 mL	590	10	29.7 ± 3.0	4.743	3.193	71.85	67.1	32.9	2.20 ± 0.15	0.8 ± 0.06
**S8**	ethyl acetate 11 mL + methanol 3 mL	550	05	25.9 ± 2.6	4.742	3.183	71.60	67.5	32.5	1.9 ± 0.13	1.8 ± 0.13
**S9**	ethyl acetate 11 mL + methanol 3 mL	500	05	15.4 ± 1.5	4.740	3.180	71.49	66.3	33.7	1.7 ± 0.12	2.3 ± 0.16

To further support the optimization strategy, additional explored deposition conditions are listed in Table S1, with the corresponding transmittance and reflectance spectra shown in Figure S6. These supplementary samples (SS1–SS10) represent a broader parameter space explored during this study and highlight the strong influence of solvent chemistry, precursor concentration, and misting time on film thickness, resistivity, and optical transparency. For example, highly polar protic solvents such as ethanol (SS1) and water (SS3) resulted in significantly higher resistivity due to rapid hydrolysis, while nonpolar solvents produced lower transmittance or unstable film growth. This further highlighted the importance of using an aprotic solvent such as ethyl acetate in combination with methanol in controlled ratios to regulate the hydrolysis process and achieve balanced optoelectronic performance discussed in this manuscript.

When depositions were carried out, the laboratory room temperature was 23–26 °C, which influenced the total time required for the misting of solution. A volume of 14 mL of ethyl acetate solution required approximately 10 min to mist, and this duration was therefore taken as the reference misting time. Half of the solution was used for the deposition of all films (5 min misting time), with the exception of sample **S7**, for which the entire solution was misted (10 min misting time). These systematic variations allowed us to assess the combined effects of solvent composition, deposition temperature, and misting time on the growth behavior and resulting functional properties of the films.

Residual chlorine was detected in the films by both XPS and EDS analysis. This incorporation is attributed to the use of SnCl_4_ as the precursor. The content of Cl in the films varied with solvent type, misting time, and deposition temperature, which can influence the kinetics of precursor deposition and film growth. The presence of residual chlorine when SnCl_4_ was used as a precursor has been reported in the literature.^16,17^

The X-ray diffraction (XRD) patterns of the as-deposited films, shown in Figure 2, confirmed that the films were phase-pure and crystallized in the cassiterite structure (SnO_2_) with a tetragonal rutile-type lattice (space group *P*4_2_/*mnm*). The absence of any secondary phases or impurity peaks indicated that high-purity SnO_2_ films were successfully deposited under the selected conditions. All films exhibited a polycrystalline nature, with clear diffraction peaks located at approximately 26.5°, 33.9°, 37.9°,51.7°, 54.7°, 61.9°, 64.8°, and 65.9°, corresponding to the (110), (101), (200), (211), (220), (310), (112), and (301) reflections, respectively. These diffraction values were indexed to ICSD # 1526637 (*P*4_2_/*mnm* space group, *a* = *b* = 4.736 Å, *c* = 3.201 Å, *V* = 71.812 Å^3^), as shown in Figure 2.

**Figure 2:**
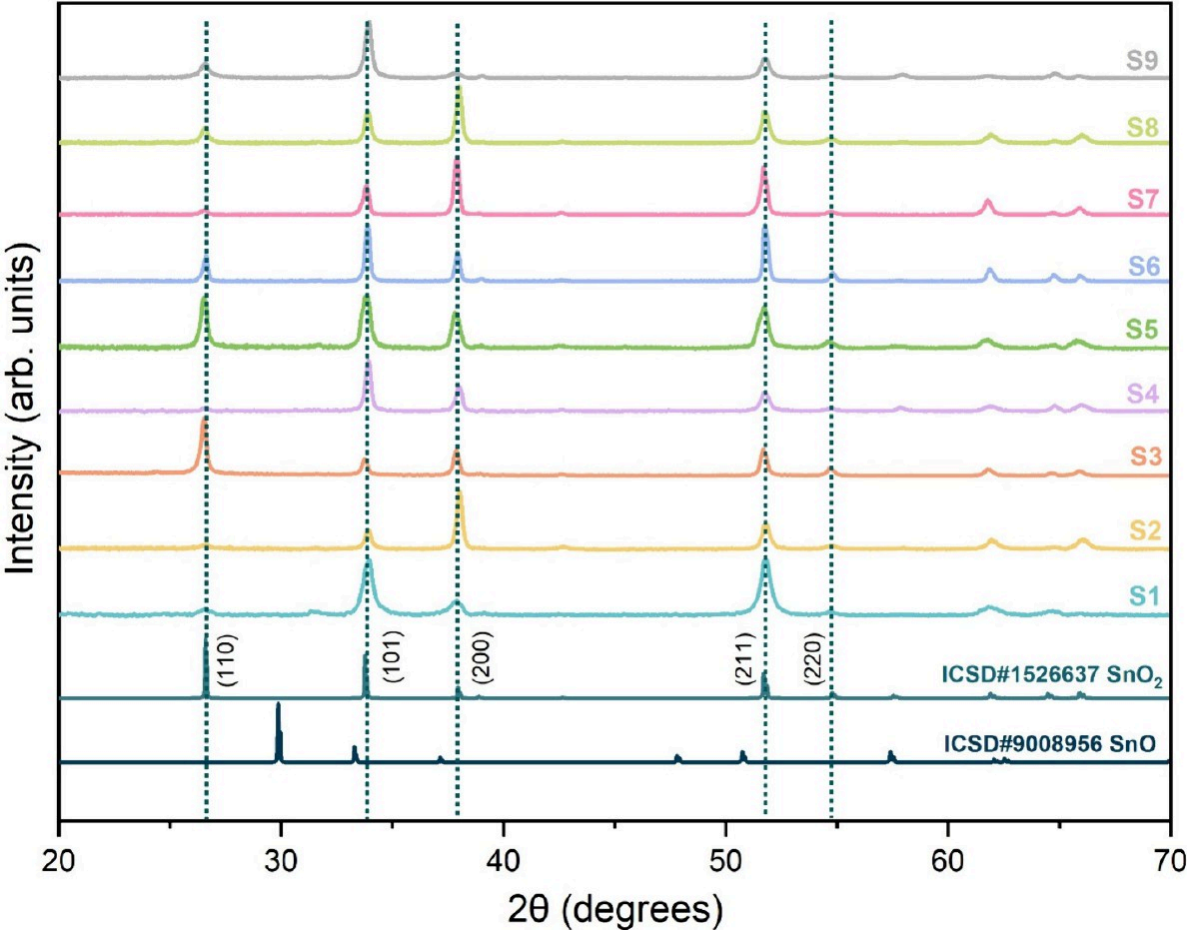
Collated XRD diffractograms of SnO_2_ films.

The crystallite sizes (*D*) were determined through the Scherrer formula,^18^ as shown in eq 1:

1 $$D=\frac{k\lambda }{\beta \cos\theta_{B}}$$

The calculated crystallite size varied between 12.7 and 31.7 nm, highlighting the significant influence of solvent choice, deposition temperature, misting time, and chlorine content on the microstructure of films. The film deposited using pure ethyl acetate (**S1**) showed the smallest crystallite size of 12.7 nm. This sample also exhibited the highest chlorine content (as shown in Table 1) However, the addition of cosolvents resulted in an increase in crystallite size (23.7–31.7 nm) and a corresponding substantial reduction in chlorine content (0.5–1.2 at %). This suggests that the cosolvent not only influenced precursor decomposition but also facilitated the removal of chlorine, which seems to have created an environment more suitable for crystallite growth. The largest crystallite size of 31.7 nm was achieved when MeCN (acetonitrile) was used as a cosolvent with ethyl acetate, which also showed a lower chlorine content than **S1**.

No direct correlation was found between the chlorine content and crystallite sizes, except the observation that samples with higher chlorine content had smaller crystallite sizes. This indicates that the solvent choice had a noticeable influence on the crystallite size. MeOH and MeCN possess significantly higher dielectric constants, 32.7 and 37.5 at 25 °C, respectively, compared to 6.02 at 25 °C for ethyl acetate. In the low polarity environment of ethyl acetate, the precursor was likely less dissociated and might exist as larger aggregates. This can result in less dense grain morphology, as observed in **S1**. The higher concentration of residual chlorine (9.5 atom %) in the film can also be linked to the low reactivity of the solvent.

MeOH and MeCN, being more polar solvents (high dielectric constant), can effectively solvate and dissociate SnCl_4_, which can produce films with larger grains, as observed in **S6**. The enhanced precursor–solvent interaction can also promote more efficient precursor decomposition and facilitate the removal of chlorine as a volatile byproduct. Films deposited involving MeOH and MeCN showed drastically lower chlorine content compared to the film deposited using only ethyl acetate. In mixed-solvent systems, the solvents were likely decomposed at slightly different temperatures, and water was released at different rates. These differences affected the rate of hydrolysis and condensation, which in turn influenced the crystal growth.

Similarly, when only MeOH was used as a solvent, the crystallite size was smaller compared to the mixture of solvents. SnCl_4_ hydrolyzes rapidly in methanol because of its high polarity and protic nature, which promotes fast formation of Sn–O species. Ethyl acetate, being less polar and aprotic, slows this hydrolysis and delays condensation. When used together, methanol enables efficient precursor conversion, while ethyl acetate moderates the reaction rate. This controlled hydrolysis–condensation process supported more uniform nucleation and sustained crystal growth, resulting in larger and better-ordered crystallites than either solvent alone. Mixed solvent systems (ethyl acetate–MeOH, ethyl acetate–MeCN) provided a balance between kinetic control and thermodynamic relaxation, resulting in larger and more ordered crystallites. This suggests that the interplay between physical properties of solvents and deposition conditions can govern the nucleation and growth behavior during deposition.

Keeping the solvent ratios and other deposition conditions the same, the effect of misting time on the film properties was examined. By increasing the misting time of the precursor solution from 5 min (**S2**) to 10 min (**S7**), the crystallite size (shown in Table 1) increased from 25.5 to 29.7 nm. The extended deposition period allowed continued material deposition that allowed crystallites to grow larger and form a thicker, more compact film. A noticeably larger unit cell volume (71.852 Å) was observed for **S7** compared to **S2** (71.484 Å). This difference may be attributed to the formation of a thicker film, which could lead to a more relaxed lattice structure or the incorporation of a different defect density over an extended deposition period.

The film deposited at 500 °C (**S9**) showed a crystallite size of 15.4 nm, which increased to 25.9 nm at 550 °C (**S8**) and stabilized around 25.5 nm at 590 °C (**S2**). At higher temperatures, atoms gain more thermal energy, which helps them overcome diffusion barriers. As a result, they can move to more stable lattice sites, which promotes the growth of larger and more ordered crystallites. This trend is common in thin film growth, and similar findings for SnO_2_ films deposited via AACVD have been reported previously.^3^

This inverse relationship is found between the chlorine content and the deposition temperature, which can be attributed to the thermal energy available during the pyrolysis process. At the highest temperature of 590 °C, sufficient energy was supplied to enable complete decomposition of the SnCl_4_ precursor. As a result, chlorine was efficiently volatilized and removed from the film as a byproduct, such as HCl. When the temperature was reduced to 550 °C or more noticeably to 500 °C, the available thermal energy might become insufficient to fully enable the decomposition process. Overall, the films (**S1** and **S9**) with the highest Cl impurity showed the smallest crystallite sizes, which might have induced lattice distortion. A similar broadening of XRD peaks with increasing impurity concentration has also been reported by Krishnakumar et al.^19^ Chlorine that originated from incomplete decomposition of the SnCl_4_ precursor can remain either at grain boundaries or partially substitute oxygen sites in the SnO_2_ lattice. The presence of chlorine at grain boundaries disrupted the crystal lattice and acted as a pinning center, inhibiting grain boundary migration and coalescence during growth. As a result, chlorine did not promote grain growth but instead hindered it by increasing structural disorder and reducing grain boundary mobility, which in turn affected both the structural quality and the electrical properties of the films.

The lattice parameters and unit cell volumes were found to be close to those of standard bulk rutile SnO_2_, indicating that all films were in the rutile phase and were largely unstrained. Although the overall crystal structure remained consistent across the samples, slight variations in the lattice parameters may be attributed to subtle factors such as oxygen vacancies, chlorine incorporation, interstitial atoms, or differences in thermal expansion during deposition. The correlation between lattice parameter *a* and chlorine content in the films is presented in Figure S2. The calculated preferred orientations for all samples are presented in Figure S1, and the effects of the deposition temperature and solvent type on these orientations are discussed in the Supporting Information.

The data strongly indicate that the use of solvent mixtures, along with variations in their ratios, had a significant influence on the film properties. These mixtures not only led to a substantial reduction in chlorine impurities but also promoted optimized crystallite growth and a preferred orientation. As a result, films produced with mixed solvents exhibited superior characteristics compared to those deposited by using pure solvents alone. This demonstrates that the film’s microstructure and composition can be finely tuned through precise control of the solvent system.

X-ray photoelectron spectroscopy (XPS) was used to analyze the surface chemical composition, electronic states, and quantification of the different elements present in the tin oxide films. The deconvoluted XPS spectra of Sn 3d, O 1s, and Cl 2p are shown in Figure S4. The Sn 3d spectra of all of the samples are shown in Figure 3a. The binding energy values of Sn 3d_5/2_ were found between 486.5 and 486.9 eV, close to the value typically reported for stoichiometric SnO_2_ (486.6 eV). The spin–orbit splitting between Sn 3d_5/2_ and Sn 3d_3/2_ was found to be 8.4 eV.^20,21^ No contributions were observed from Sn­(II), which typically appears at slightly lower binding energy (486 eV).^19^

**Figure 3:**
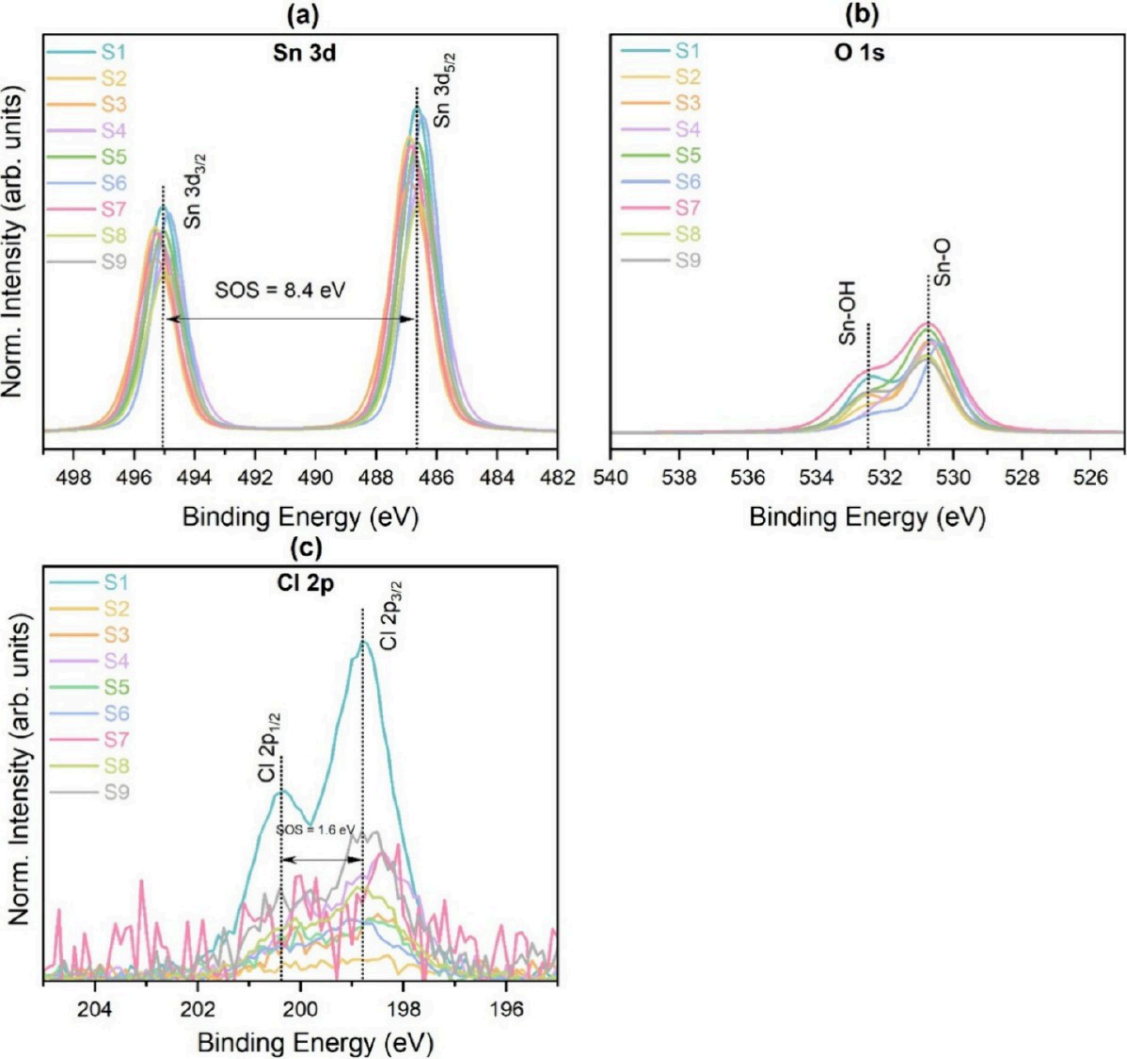
XPS spectra of (a) Sn 3d, (b) O 1s, and (c) Cl 2p.

The Cl 2p spectra (Figure 3c) were analyzed to identify the chemical state of residual Cl in the films. A prominent doublet corresponding to the Cl 2p_3/2_ and Cl 2p_1/2_ peaks was observed. The Cl 2p_3/2_ peak was found at a binding energy of approximately 198.7 eV. This is consistent with chlorine existing in a chloride-like state, likely as an impurity at the grain boundaries or substituting oxygen within the SnO_2_ lattice.^20^ The spin–orbit splitting value for this doublet was determined to be 1.6 eV.

The O 1s spectra (Figure 3b) were deconvoluted into two components to determine the bonding states of different oxygen present in the samples. The first peak centered at approximately 530.8 eV was attributed to lattice oxygen corresponding to Sn–O bonds. However, the higher binding energy peak at around 532.2 eV was assigned to surface hydroxyl groups (−OH).^22^ The relative atomic percents of both types of oxygens are shown in Table 1.

The observed shift in binding energy of the Sn peaks can be rationalized by considering the competing influences of oxygen vacancies, surface hydroxylation, residual chlorine, and changes in the local Sn–O bonding environment. Oxygen vacancies act as donor defects, increasing electron density at Sn sites, which can shift the core-level binding energy to a lower value. However, higher surface hydroxyl content, increased oxygen stoichiometry, and the presence of electronegative species such as chlorine can withdraw electron density from Sn and, thereby, can shift the binding energy to higher values. It has been reported by Jeon et al. that the incorporation of halogen atoms into SnO_2_ lattice resulted in a noticeable shift of the Sn 3d core-level peaks toward higher binding energies.^21^ Akbar et al. have reported that zinc incorporation in SnO_2_ altered the electronic environment of tin due to the generation of oxygen vacancies. These vacancies introduced additional electrons, which caused the Sn 3d core-level peaks to shift to lower binding energies.^22^

Table 1 shows the relative atomic % of tin oxide and hydroxide, the O/Sn ratio, and the atomic % of residual chlorine present in each sample. The lowest binding energy values were observed for **S6** and **S4** (∼486.5 eV). For **S4**, this shift to lower binding energy could be related to oxygen vacancies as it showed a substoichiometric O/Sn ratio of 1.3. Similarly, **S6** also exhibited a substoichiometric O/Sn ratio of 1.4.

However, the highest positive shifts were observed for **S2**, **S3**, and **S7** (∼486.8 eV) having relatively low chlorine content of 0.5 and 0.8 at %, respectively. **S7** appeared to exhibit an oxygen rich surface with an O/Sn ratio of 2.2 and significant surface hydroxylation (32.87%).

Interestingly, **S1** and **S5** showed very similar binding energies of 486.65 eV, which is close to the expected stoichiometric value. However, their chlorine levels and O/Sn ratios are very different. **S1** has a high chlorine content of 9.5 atom % and an O/Sn ratio of 2.08. **S5** has low chlorine at 0.8 atom % but a higher O/Sn ratio of 2.1. This shows that multiple factors interact and balance each other. For example, the strong electron-withdrawing effect of chlorine in **S1** might be balanced by surface hydroxylation. The observed binding energy shifts could be a complex interplay among surface stoichiometry, hydroxylation , and the presence of residual chlorine. The high O/Sn ratio observed for **S5** is better attributed to hydroxylation and surface chemistry effects associated with the MeOH-only solvent system (rapid hydrolysis) rather than to chlorine incorporation alone.

The scanning electron microscopy (SEM) and energy dispersive X-ray spectroscopy (EDS) images are presented in Figure 4. SEM provided crucial insights into the surface morphology, grain shape, size, packing density, and overall uniformity of the as-deposited SnO_2_ films. The presence of chlorine in the samples was also confirmed by EDS.

**Figure 4:**
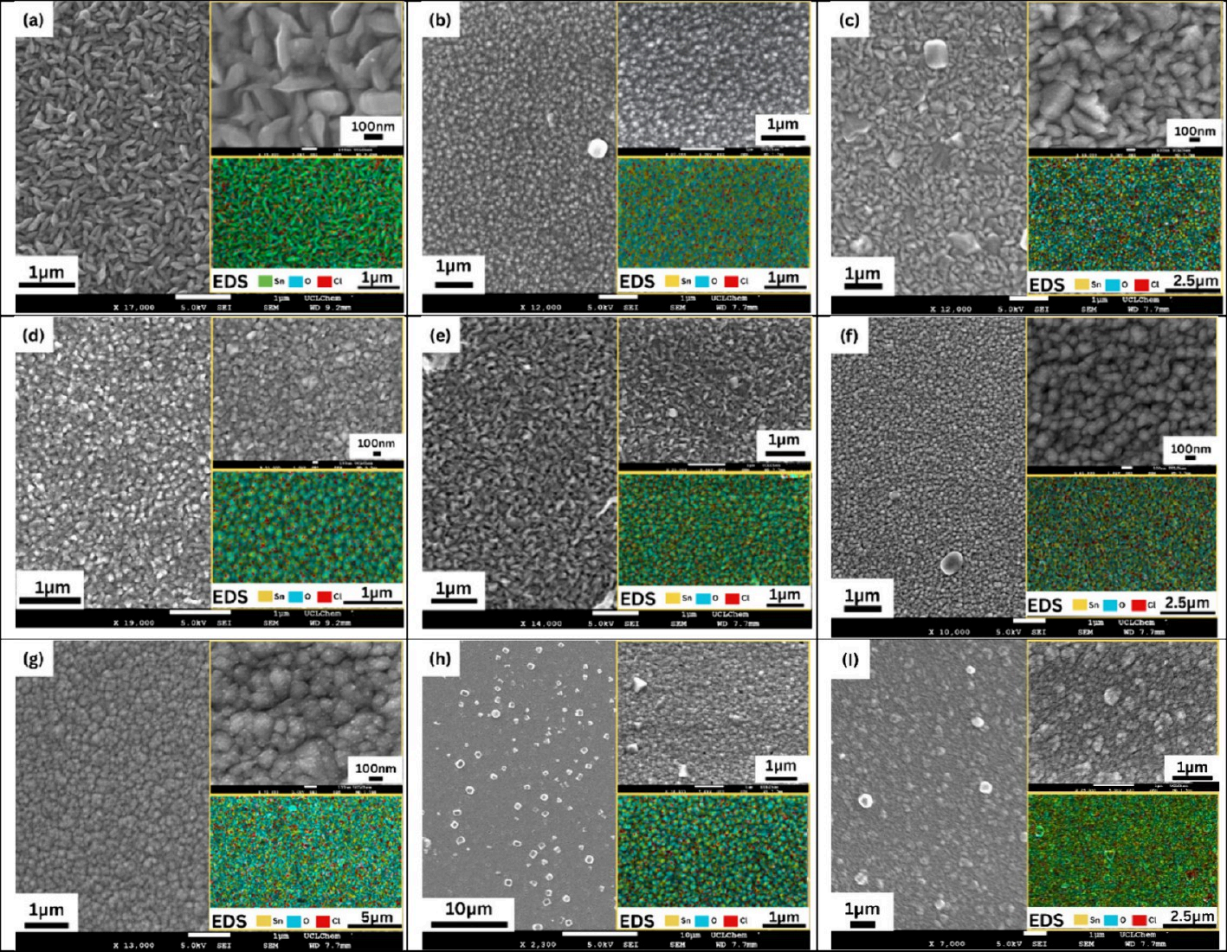
SEM and EDS images of (a) **S1**, (b) **S2**, (c) **S3**, (d) **S4**, (e) **S5**, (f) **S6**, (g) **S7**, (h), **S8**, and (I) **S9**.

The film deposited with pure ethyl acetate (Figure 4a) showed distinct, relatively elongated, and somewhat faceted grains. While the crystallite size determined by XRD for **S1** was the smallest at 12.7 nm, the SEM image suggests that these smaller crystallites may aggregate into larger and visible features. The film appeared less dense compared to films prepared with cosolvents, with some spaces visible between the elongated particles. This morphology correlated with the preferential growth along the (101) and (211) planes, as indicated by XRD texture analysis.

A notable transition in morphology was induced with the introduction of methanol. Sample **S2** (ethyl acetate 11 mL + MeOH 3 mL Figure 4b) exhibited a remarkably dense and uniform film, characterized by much smaller, more homogeneous, and somewhat rounded grains. This denser packing was observed despite **S2** having a larger crystallite size (25.5 nm) compared to that of **S1**, suggesting that the methanol facilitated a more efficient packing of the growing SnO_2_ crystallites. Similarly, **S3** (ethyl acetate 7 mL + MeOH 7 mL, Figure 4c) showed a compact surface of well-merged, polygonal faceted grains. The crystallite diameter increases further to 28.1 nm and the (200) texture remains pronounced. **S4** (ethyl acetate 3 mL + MeOH 11 mL, Figure 4d) showed a very dense film composed of relatively uniform, faceted, and somewhat squarish grains, morphologically resembling **S2** and **S3** in terms of overall density and packing. These films consistently exhibited larger crystallite sizes (23.7–28.1 nm) and a strong (200) preferred orientation, indicating that the solvent mixture promoted both enhanced crystallite growth and specific crystallographic alignment.

The film **S5** deposited using only methanol (Figure 4e) showed relatively uniform, somewhat flattened or tabular-shaped grains that were densely packed, resulting in a smooth and continuous film appearance. This morphology agrees with the calculated crystallite size (19.7 nm from XRD) and a dominant (200) orientation. This suggested that while MeOH facilitated good packing and (200) growth, the presence of ethyl acetate in mixtures further enhanced the crystallite size.

The **S6** film presented a distinct morphology, characterized by very fine, highly uniform, and densely packed rounded grains. Despite visually appearing fine-grained, it had the largest crystallite size (31.7 nm) among all samples, as determined by XRD. The effect of solvent on the morphology of SnO_2_ films has already been studied.^3^ When the misting time was increased from 5 min (**S2**) to 10 min (**S7**), the film morphology remained generally dense and uniform, consistent with the maintained (200) preferred orientation. However, the grains in **S7** appeared slightly larger and possibly more fused, reflecting the increased accumulation of material on the substrate surface. This morphological evolution is consistent with the observed increase in crystallite size from 25.5 nm for **S2** to 29.7 nm for **S7**, where an extended misting time might have allowed existing crystallites to grow larger. A denser surface morphology with increased deposition time or film thickness has already been reported in SnO_2_ films.^23^

The deposition temperature also significantly impacted the morphology of the films. A clear shift in morphology was observed, from a dense and uniform film at 590 °C (**S2**) to a sparser distribution of discrete grains on a less continuous layer at 550 °C (**S8**). At 500 °C (**S9**), the film appeared more porous and less continuous. These findings suggested that below approximately 550 °C, growth becomes kinetically controlled, favoring different crystallographic planes and leading to less continuous film morphology. Similar results have already been reported.^24^

The SEM analysis clearly demonstrated that the film morphology of SnO_2_ can be precisely controlled by controlling the solvent composition, misting time, and deposition temperature. A control over crystallographic structure and surface morphology holds significant importance for the functional properties of SnO_2_ films, such as their gas sensing performance (which benefits from optimized grain size and accessible surface area)^25^ and electrical conductivity.

While SEM revealed the overall grain structure and film coverage, atomic force microscopy (AFM) was used to study the nanoscale surface roughness of the SnO_2_ films. The quantitative comparison of topographic variations across different samples was made, and the average root-mean-square (RMS) roughness values are summarized in Table 2. The corresponding AFM images of all of the samples are shown in Figure 5.

**Figure 5:**
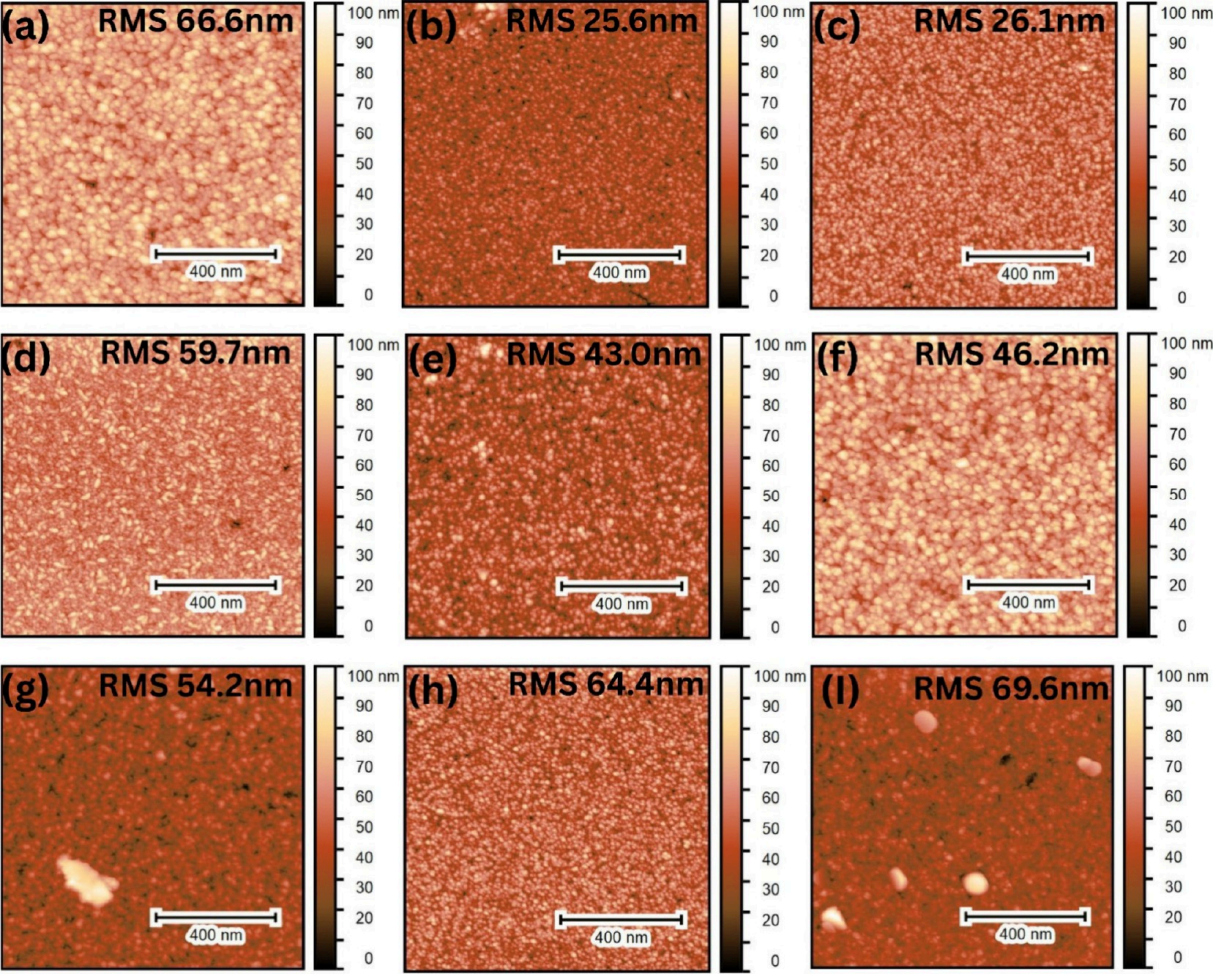
AFM images of (a) **S1**, (b) **S2**, (c) **S3**, (d) **S4**, (e) **S5**, (f) **S6**, (g) **S7**, (h) **S8**, and (I) **S9**.

**Table 2:** Film Thickness, Electrical Properties, Calculated Sheet Resistance, Figure of Merit (F.O.M.), Band Gap, Transmittance at 550 nm, and Average Transmittance across the Visible (380–750 nm), Near-Infrared (750–1400 nm), and Short-Wave (1400–2500 nm) Regions and Average RMS Roughness Values (± Standard Error) of SnO_2_ Films and TEC-15

film	film thickness (±30 nm)	resistivity (Ω cm)	mobility (cm^2^ V^–1^ s^–1^)	carrier concentration (cm^–3^)	sheet resistance (Ω □^–1^)	F.O.M. (Ω^–1^)	band gap (eV)	*T*_λ550_ (%)	*T*_λ380–750_ (%)	*T*_λ750–1400_ (%)	*T*_λ1400–2500_ (%)	average R.M.S. roughness (nm)
**S1**	547	2.9 (±0.9) × 10^–3^	14.3	1.5 × 10^20^	53.0	7.4 × 10^–4^	3.81 (±0.02)	72.3 (±0.5)	70.3	74.5	54.0	66.6 ± 2.3
**S2**	500	9.6 (±0.5) × 10^–4^	22.5	2.9 × 10^20^	19.2	6.4 × 10^–3^	3.84 (±0.01)	81.1 (±0.6)	77.8	75.0	66.6	25.6 ± 0.9
**S3**	463	8.5 (±0.7) × 10^–4^	23.0	3.2 × 10^20^	18.4	6.0× 10^–3^	3.97 (±0.01)	80.2 (±0.6)	79.0	82.31	71.2	26.1 ± 0.5
**S4**	400	1.3 (±0.5) × 10^–3^	18.9	2.6 × 10^20^	32.5	3.7 × 10^–3^	3.80 (±0.01)	81.0 (±0.5)	79.4	74.3	65.4	59.7 ± 14.5
**S5**	474	1.7 (±0.6) × 10^–3^	24.0	1.5 × 10^20^	35.9	2.0 × 10^–3^	3.82 (±0.03)	77.0 (±0.5)	77.7	73.3	63.6	43.0 ± 9.9
**S6**	549	2.7 (±0.6) × 10^–3^	14.3	1.6 × 10^20^	49.2	1.3 × 10^–3^	3.82 (±0.02)	75.7 (±0.6)	73.8	78.3	78.05	46.2 ± 1.9
**S7**	1000	1.2 (±0.8) × 10^–3^	25.1	2.1 × 10^20^	12.0	1.2 × 10^–3^	3.93 (±0.04)	65.4 (±0.6)	64.2	60.1	13.9	54.2 ± 7.2
**S8**	444	2.0 (±0.5) × 10^–3^	15.8	1.9 × 10^20^	45.0	4.8 × 10^–3^	3.82 (±0.01)	85.7 (±0.4)	79.5	75.8	56.2	64.4 ± 12.1
**S9**	375	5.9 (±0.4) × 10^–3^	15.6	6.8 × 10^19^	157.3	9.0 × 10^–4^	3.79 (±0.01)	82.3 (±0.5)	78.7	75.2	51.8	69.6 ± 13.3
TEC-15	340	4.5 × 10^–4^	34.0	4.0 × 10^20^	13.2	1.17 × 10^–2^	3.97	83.1	82.4	73.3	24.5	–

The RMS roughness values spanned 25–69 nm, with **S2** and **S3** exhibiting the smoothest surfaces (25.6 and 26.1 nm, respectively). Their low roughness is closely related to the uniform and dense morphology observed by SEM and larger crystallites, as determined by XRD and reduced Cl and surface hydroxyl content by XPS. In contrast, films deposited using single solvents exhibited higher roughness, i.e., **S1** (66.6 nm) and **S5** (43.0 nm), highlighting the influence of precursor chemistry and grain packing on nanoscale topography.

Lowering the deposition temperature from 590 °C (**S2**) to 550 °C (**S8**) and 500 °C (**S9**) progressively increased roughness to 64.4 and 69.6 nm, respectively, reflecting reduced surface diffusion and kinetically limited growth. Similar observations are already reported for SnO_2_ films.^26^ Similarly, extending the misting time increased the roughness, as observed for the thicker film **S7** (54.2 nm).

Similar roughness values for SnO_2_ films are widely reported in the literature.^27–29^ RMS roughness around 20 nm is often considered optimal for SnO_2_-based TCO and electron transport layer (ETL) applications, balancing light scattering with smooth charge transport. Hoang Huy and Bark^28^ showed that increased roughness in SnO_2_ improved solar performance by enhancing light scattering, while Juraić et al.^29^ linked SnO_2_ roughness to diffuse scattering beneficial for photovoltaic. The optimized films, particularly **S2** and **S3**, thus lie in this favorable regime.

**S1** (ethyl acetate only, 590 °C) also showed a markedly rougher surface (66.6 nm), consistent with the SEM evidence of elongated, less dense packing. The MeOH-only film (**S5**) showed moderate roughness (43.0 ± 9.9 nm) but with a large standard error, pointing to lateral heterogeneity across the scanned areas. In contrast, **S6** (ethyl acetate–MeCN, 590 °C) showed intermediate roughness (46.2 nm), indicating that crystallite size alone (31.7 nm, XRD) did not set the nanoscale topography. This indicated that the nanoscale topography was governed not only by deposition temperature but also by the precursor solution chemistry.

The optical properties of the SnO_2_ films were determined by using UV–vis spectroscopy. The transmittance and reflectance spectra of all of the samples are shown in Figure 6. All the films exhibited high optical transparency ranging from 72.3% to 85.7% for films deposited with a 5 min misting time.

**Figure 6:**
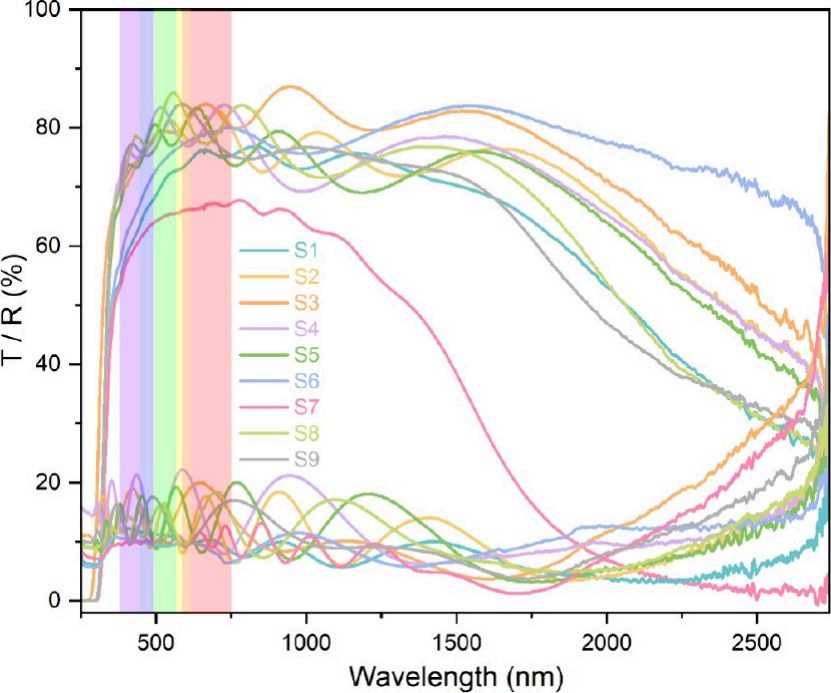
Transmittance (*T*) and reflectance (*R*) of all of the samples.

The highest transmittance was observed for **S8** (85.7%), which was deposited at 550 °C, followed by **S9** (82.3%) and **S2** (81.1%), indicating the role of deposition temperatures on optical clarity. This observation agrees with the reported literature where transmittance was found to decrease with the deposition temperature and has been attributed to increased film thickness, surface roughness, or enhanced absorption due to free charge carriers.^24,30,31^ **S2** exhibited higher carrier concentration (2.9 × 10^20^ cm^–3^) and lower transmittance (81.1%) than **S8** and **S9** (precursor chemistry same as **S2**). However, other reports have shown an increase in transmittance as the deposition temperature was increased, and this has been attributed to increased film crystallinity and reduced defect-related scattering.^32,33^

The choice of solvent also had an impact on the optical properties of the SnO_2_ films. In general, films deposited using an ethyl acetate/MeOH solvent system showed higher transmittance than the film deposited using ethyl acetate/MeCN. For instance, despite having similar thickness, **S2** showed markedly higher transmittance (81.1%) than **S6** (75.7%). This suggests that ethyl acetate/MeOH might have promoted smoother surface morphology, lower defect density, and reduced light scattering losses. Furthermore, the ethyl acetate to MeOH ratio was also important.

Films deposited by using an ethyl acetate–methanol mixture showed higher transmittance than films deposited by using either solvent alone. However, the film deposited with the same ethyl acetate:MeOH ratio as of **S2** but increased misting time (**S7**, 10 min misting time) showed a decrease in *T*_λ550_ (65.4%), which can be associated with increased thickness and scattering, as well as increased free-carrier absorption. **S3** showed a transmittance of 80.2% at 550 nm, consistent with its mixed solvent composition and balanced film morphology.

The observed variation in transmittance with different solvents agrees with previous studies, which have reported that solvent choice can strongly affect the optical properties of SnO_2_ thin films through changes in microstructure and defect states.^3,34,35^

**S1** exhibited a relatively low *T*_λ550_ of 72.3%. This diminished transparency can be directly linked to its higher thickness and residual chlorine content (9.5 at %). The presence of such a high concentration of impurity scattering centers can effectively reduce overall light transmittance.^2,20,36^ Although **S2** and **S6** had similar O/Sn ratios, **S6** showed lower transmittance due to its greater film thickness and higher surface roughness. Solvent-dependent changes in microstructure and free charge carrier concentrations also had an impact, suggesting that optical transparency depended on several factors and not stoichiometry alone. The transmittance and reflectance spectra of commercial FTO (TEC-15, NSG Pilkington) and uncoated float glass are shown in Figure S3. TEC-15 showed a transmittance of 84.3% at 550 nm, as shown in Table 2.

Across all films, a drop in transmittance was observed for near-infrared (NIR, 750–1400 nm) and short-wave infrared (SWIR, 1400–2500 nm) regions, which is a characteristic of functional TCO materials and is attributed to free-carrier absorption (Drude effect).^2,36^ For most samples, the average NIR transmittance is higher than the SWIR transmittance (Table 2), showing useful IR selectivity.

For TCO applications in photovoltaics and smart windows that require light transmission up to 1.3 μm, **S3**, **S2**, and **S8** can be the best choices as they showed high visible and NIR transmittance and practical sheet resistance.^37^ Such a property of NIR reflection or heat shielding can find applications in photovoltaics, smart windows, or heat mirrors.^37–39^ For heat-shielding glazing or heat-mirror coatings, where SWIR blocking is desired, **S7** can be a strong candidate.^37^

The strong UV absorption exhibited by all of the films suggests their potential as functional coatings in smart windows and display panels to protect sensitive materials or human eyes from harmful UV rays.

Figure 7 shows the haze spectra of all of the samples. Sample **S7** showed the highest haze followed by **S1**, which is consistent with their higher thickness and surface roughness. In contrast, films such as **S2**, **S3**, **S4**, **S5**, **S6**, **S8**, and **S9** showed comparatively lower haze values (below 10%), particularly across the visible region. For all samples, the haze decreased progressively toward longer wavelengths, indicating reduced light scattering in the near-infrared region. These trends correlated well with the AFM and thickness results, suggesting that smoother and thinner films contributed to improved optical clarity.

**Figure 7:**
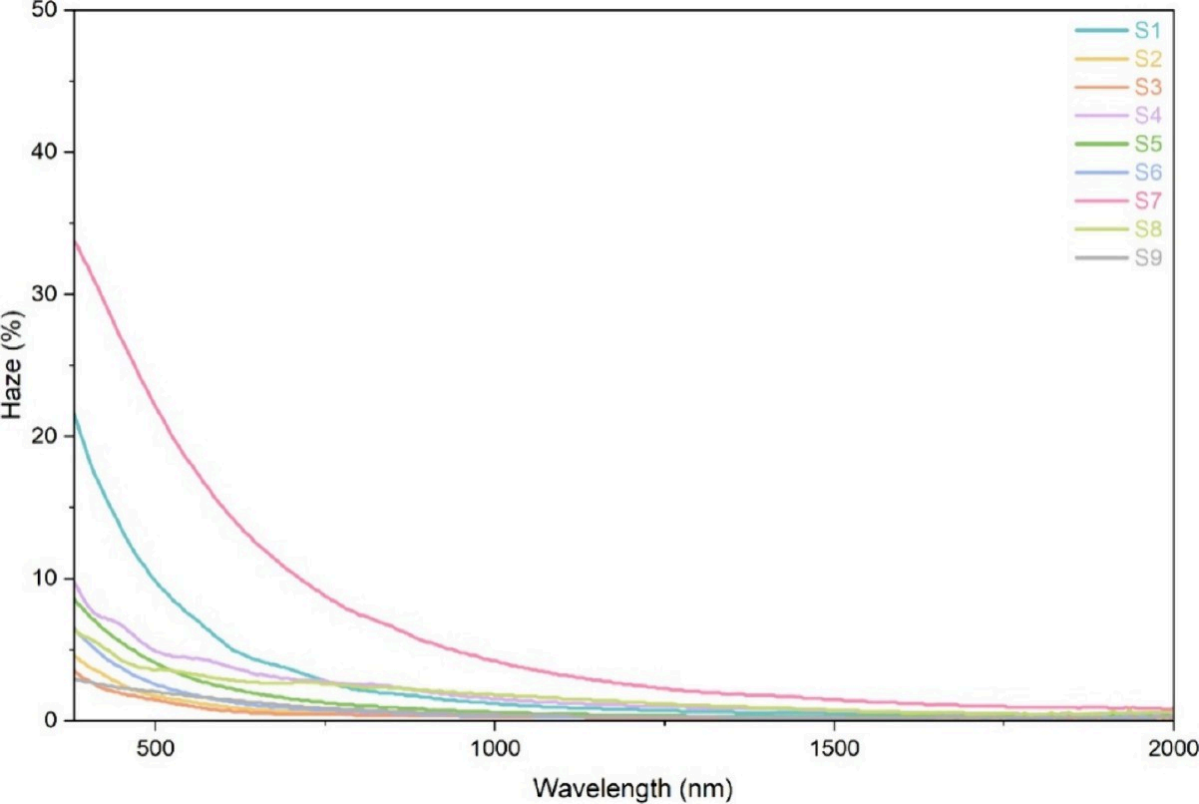
Haze spectra of all of the samples.

The optical band gap (*E*_g_) was determined using the Tauc method assuming a direct allowed transition and is shown in Figure 8. In this method, the linear portion of a plot of (α*hν*)^2^ versus photon energy *E*_g_ (eV) near the high-energy absorption edge was extrapolated to the energy axis to estimate the band gap. In the Tauc relation (eq 2),^40^ α is the absorption coefficient, *A* is a proportionality constant, and *hν* represents the photon energy, where *h* is Planck’s constant and *ν* is the frequency of the incident radiation.

2 $${\left(\alpha h\nu \right)}^{2}=A\left(h\nu -E_{g}\right)$$

**Figure 8:**
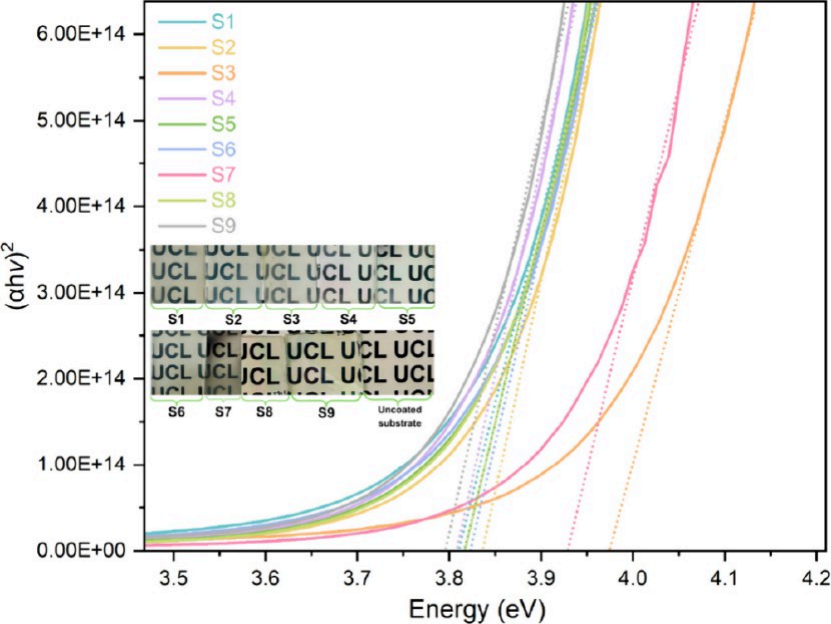
Tauc plots and photographs of all of the films and uncoated substrate.

The optical band gap values lied within the narrow range of 3.79–3.97 eV. Similar band gap values have been widely reported for SnO_2_ films.^3,34,35^ Samples **S2** and **S3** exhibited slightly wider band gaps (3.84–3.97 eV), which correlated with their lower thickness, higher carrier concentrations, and improved crystallinity. The combination of a thinner film and a dense, uniform morphology likely reduced defect-related absorption, shifting the absorption edge to higher energies (Moss–Burstein effect).^3^ Similar results have been widely reported in literature.^41,42^

The lowest band gap of 3.79 eV was measured for **S9**, which also shows the lowest carrier concentration and highest resistivity in the series. The defects such as oxygen vacancies or residual chlorine are known to create midgap states that lead to a red shift of the absorption edge, narrowing the measured band gap.^25^

Overall, the data confirmed that the band gap variation in these SnO_2_ films was mainly influenced by the interplay of film thickness, defect-related states, and carrier concentration rather than by intentional dopant effects. Thinner and more compact films generally exhibited a slight blue shift, while thicker or more defective films showed red shift.

The band gaps were also calculated using the Kubelka–Munk (K-M) function from diffuse reflectance data, as shown in Figure S5, with the values reported in Table S1.

Hall effect measurements were performed at room temperature using a Van der Pauw configuration on the most conductive areas of all tested films to evaluate their electrical conductivity. The results are presented in Table 2. The negative Hall coefficient values confirmed the n-type conductivity of the films. The films were stored in air under ambient conditions for 18 months, during which no noticeable film degradation or measurable changes in electrical properties were observed. The films (**S2** and **S3**) were subjected to 20 cycles of Scotch tape testing, during which no noticeable edge peeling, delamination, or surface degradation was observed. Electrical measurements performed after the tape tests showed no measurable changes in the resistivity, indicating that the electrical performance of the films was preserved. In addition, the films demonstrated good resistance to mechanical damage during manual scratching tests by using a stainless-steel spatula.

It is well-known that the physical properties of oxides are strongly influenced by deviations from stoichiometric composition (native disorder) and the nature and concentration of foreign atoms incorporated into the crystal lattice. Since tin oxide is an n-type semiconductor, oxygen vacancies (V_o_) or interstitial tin atoms (Sn_i_) are expected to act as donors in pure SnO_2_.^25,43^ The SnO_2_ films appeared to be oxygen-deficient (Table 1). According to the literature, the formation of oxygen vacancies on the surface and subsurface regions of growing SnO_2_ occurs under the applied deposition conditions, specifically at a high temperature of above 500 °C.^44^

The lowest resistivity of 8.5 × 10^–4^ Ω cm was recorded for **S3**, which also exhibited one of the highest carrier concentrations (3.2 × 10^20^ cm^–3^) and high mobility (23.0 cm^2^ V^–1^ s^–1^). This confirms that the mixed ethyl acetate–methanol system (7:7 mL) produced a film with reduced grain-boundary scattering and low defect density, in agreement with its compact morphology and relatively large crystallite size (from XRD).

Films deposited from the same precursor solution but with shorter (**S2**) or longer (**S7**) misting durations showed measurable differences. Extending the misting time from 5 min (**S2**) to 10 min (**S7**) increased the film thickness and crystallite size but did not lead to further improvement in conductivity. Instead, **S7** showed slightly higher resistivity (1.2 × 10^–3^ Ω cm), indicating that beyond a certain threshold, thickness-driven grain coalescence could not compensate for increased scattering or defect incorporation. This confirmed that conductivity optimization arose primarily from controlled nucleation and stoichiometry, not merely increased material deposition.

Deposition temperature had a strong influence on the electrical properties. Keeping the solvent composition the same, the film deposited at higher deposition temperature (590 °C, **S2**) exhibited lower resistivity than the films deposited at 550 °C (**S8**) and 500 °C (**S9**). The highest resistivity (5.9 × 10^–3^ Ω cm) of **S9** was accompanied by the lowest carrier concentration (6.8 × 10^19^ cm^–3^) and smallest crystallite size (15.4 nm). The higher resistivity at lower deposition temperature could be attributed to suppressed crystallite growth, reduced long-range order, and incomplete precursor decomposition at lower thermal energy, which limited the removal of chlorine impurities and resulted in higher defect scattering. A decrease in resistivity with the deposition temperature has already been widely reported.^31,32^

Similarly, the influence of the solvent type and composition on electrical properties was also evident. Films deposited using ethyl acetate alone (**S1**) or methanol alone (**S5**) exhibited higher resistivity than the mixed-solvent films. This indicates that the mixed-solvent system yielded a microstructure with fewer scattering centers, consistent with larger crystallites and lower residual chlorine content. The film deposited using ethyl acetate–MeCN (**S6**) showed intermediate resistivity (2.7 × 10^–3^ Ω cm), confirming that solvent polarity and precursor–solvent interaction played a key role in determining charge-transport pathways.

Furthermore, even within the ethyl acetate/MeOH series, small adjustments in the solvent ratio produced clear differences in electrical performance. The higher ethyl acetate fraction in **S2** (11:3) enabled moderated precursor hydrolysis, which supported dense grain packing, low chlorine incorporation, and consequently low resistivity. Increasing the MeOH content to a 1:1 ratio in **S3** (7:7) further improved conductivity, giving the lowest resistivity and highest mobility in the series, indicating that the balance between the solvent polarity and reaction rate was optimal in this case. However, further increasing the MeOH proportion in **S4** (3:11) did not yield additional improvement, suggesting that excessively fast precursor decomposition reduced the structural control and introduced more scattering sites. These trends confirm that the solvent ratio directly influenced crystallite growth kinetics, chlorine removal efficiency, and defect formation, all of which collectively governed the final electrical behavior.^3,34^ Figure 9 compares the optoelectronic performance of the films from this work with widely reported TCOs. The films are intentionally undoped, yet their performance is comparable to optimally doped films.

**Figure 9:**
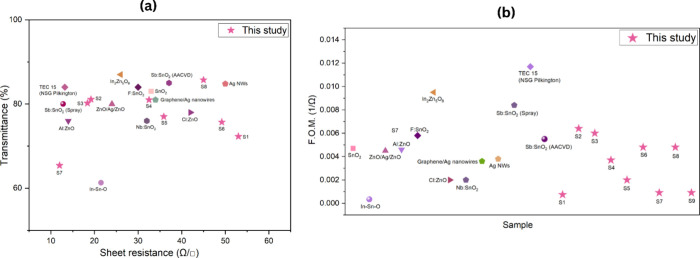
Performance comparison between the samples prepared in this work and widely reported TCO materials from the literature: (a) optical transmittance versus sheet resistance; (b) comparison of the Haacke figure of merit (references: SnO_2_,^53^ In–Sn–O,^54^ ZnO/Ag/ZnO,^55^ Al:ZnO,^56^ F:SnO_2_,^57^ In_2_Zn_5_O_8_,^58^ Cl:ZnO,^59^ Nb:SnO_2_,^60^ graphene/Ag nanowires,^61^ Ag NWs,^62^ Sb:SnO_2_,^63,64^ TEC-15 (NSG Pilkington)).

The low resistivity of SnO_2_ films due to oxygen vacancies has been widely reported in the literature.^25,45–47^ Mukhamedshina et al.^45^ reported a decrease in surface resistance when films were treated with hydrogen plasma, attributing this effect to the presence of oxygen vacancies. In contrast, an increase in the surface resistance was observed after treatment with oxygen plasma, which was attributed to the filling of oxygen vacancies. This suggests that oxygen vacancies contributed to achieving low resistivity values.^45^ In line with these reports, **S3** and **S2**, which exhibited oxygen vacancies (O/Sn ratio of 1.4), had lower resistivity compared to more stoichiometric films (**S1** and **S5**). However, higher resistivity was observed for **S4** and **S6**, which showed higher oxygen vacancies.

It also has been reported that excessive oxygen vacancies can decrease conductivity due to enhanced scattering at grain boundaries.^19,21,48^ This indicated that resistivity was not determined by oxygen vacancies alone but by the complex interplay between vacancy concentration, residual chlorine incorporation, and morphology.

The trends observed in electrical properties were also consistent with the structural and surface chemical analyses. XRD showed that larger crystallite sizes and well-defined preferred orientations were facilitated at higher deposition temperatures, which resulted in lower resistivity values. XPS revealed that **S3** and **S2** had the lowest residual chlorine content and surface hydroxylation (Table 1), conditions that could maintain high electron density at Sn sites. However, films with elevated Cl content (**S1** and **S9**), showed broader XRD peaks and higher resistivity, which confirmed that residual chlorine acted as scattering centers and disrupted conduction pathways. Consistent results have also been obtained using APCVD, where an increase in deposition temperature was correlated with a reduction in residual chlorine content (1.2 at % at 380 °C decreasing to 0.8 at % at 525 °C) and are accompanied by improved electrical conductivity.^11^ Similar results were reported by Bruneaux et al. for spray-deposited SnO_2_ films.^16^

However, a decrease in the resistivity upon chlorine doping has been reported in the literature. For example, Abass achieved a sheet resistance of 16 Ω □^–1^ at 0.4 wt % Cl via spray pyrolysis,^49^ while Cheng et al. reported a minimum of 150 Ω □^–1^ for Cl-doped SnO_2_ films deposited by ALD.^50^

Quantifying the ratio of hydroxide to oxide in the thin films is an important indicator of the film quality. The role of surface hydroxylation in determining electronic properties and device performance has been highlighted in several studies. Riaz et al. recently reported that in In_2_O_3_ thin films lower hydroxylation was beneficial for device performance. They suggested that reduced hydroxyl content (28.8 at %) minimized surface trap states and enhanced carrier mobility.^51^ Similar observations have been reported for SnO_2_, where hydroxyl groups on the surface promoted electron trapping and diminished final device performance.^21^ Our results followed a similar trend, where **S2** and **S3** showed relatively low hydroxylation. Its impact on **S3** directly reflected in its superior electrical performance, with high charge carrier mobility (23.0 cm^2^ V^–1^ s^–1^) and low resistivity (8.5 × 10^–4^ Ω cm). These findings indicated that controlling surface hydroxyl and residual chlorine content was effective for optimizing conductivity and ensuring optimized device performance.^21^ It is important to note that XPS probes only the near-surface region of the films (∼5–10 nm). Therefore, the above discussion on hydroxylation represented surface chemistry rather than the bulk of the film.

Such low resistivity for SnO_2_ is comparable to that of intentionally doped SnO_2_ films, as shown in Table 3.^6,7^ However, similarly low resistivity values of the same order of magnitude have also been reported in the literature for undoped films, as shown in Table 3.^5,12^

**Table 3:** Optoelectronic Properties of Previously Reported SnO_2_ Films Synthesized Using SnCl_4_ As a Precursor

film	thickness (nm)	resistivity (Ω cm)	mobility (cm^2^ V^–1^ s^–1^)	carrier conc. (cm^–3^)	transmittance (%)	band gap (eV)	preparation method	ref.
F:SnO_2_	1000	3.71 × 10^–3^	5.81	2.89 × 10^21^	87	3.97	spray pyrolysis	^6^
Sb:SnO_2_	230	7.35 × 10^–4^	32	6.35 × 10^20^	85.5	4.2	spray pyrolysis	^7^
SnO_2_	–	8.0 × 10^–2^	∼15	∼10^20^	–	–	ALD	^13^
SnO_2_	–	2.0 × 10^–3^	6.88	6.58 × 10^20^	80	–	APCVD	^12^
SnO_2_	500	8.0 × 10^–4^	–	–	92	–	spray pyrolysis	^5^

The optimized undoped SnO_2_ films, particularly **S2** and **S3**, exhibited sheet resistance values of approximately 19 and 18 Ω □^–1^, respectively, while maintaining reasonably high optical transmittance, which is quite good for undoped tin oxide and demonstrates the strong intrinsic conductivity achievable without extrinsic dopants. For comparison, the commercial TEC-15 coating is an F-doped SnO_2_ (FTO) film and typically shows a sheet resistance of around 14 Ω □^–1^. The fact that the undoped films approach this value highlights their promising baseline performance and indicates substantial potential for further improvement since introducing suitable dopants could readily enhance carrier concentration and mobility to reach conductivity levels comparable to, or potentially exceeding, those of commercial FTO coatings.

To evaluate the optoelectronic performance of TCO materials effectively, a figure of merit (F.O.M.), proposed by Haacke in 1976,^52^ has been used as a quantitative measure to rate the performance of samples in this work. The F.O.M. (Φ) is defined by the relationship shown in eq 3:

3 $$\Phi =\frac{T^{10}}{R_{sh}}$$

where *T* is the optical transmittance at 550 nm (in decimal form) and *R*_sh_ is the sheet resistance. Among the deposited films, **S2** and **S3** exhibited the highest F.O.M. values (6.4 × 10^–3^ and 6.0 × 10^–3^ Ω^–1^, respectively), which was consistent with their low resistivity, enhanced carrier mobility, and high visible transmittance. In contrast, films with a higher residual chlorine content or increased surface roughness, such as **S1** and **S6**, showed reduced F.O.M. values, highlighting the importance of precursor decomposition and microstructural control. Although the commercial TEC-15 (F-doped SnO_2_) displayed the higher F.O.M. (1.4 × 10^–2^ Ω^–1^), the optimized undoped AACVD films approached comparable performance without intentional doping. These results demonstrated that controlled deposition parameters can enable a favorable balance between transparency and conductivity.

Figure 10 compares the figure of merit of the SnO_2_ films from this work with those of previously reported undoped SnO_2_ films deposited via AACVD for TCO applications. Among the deposited films, **S2** and **S3** showed improved overall performance compared with reported undoped AACVD SnO_2_ films, demonstrating a more favorable balance between optical transparency and electrical conductivity. This further highlights the effectiveness of the optimized deposition conditions used in this study.

**Figure 10:**
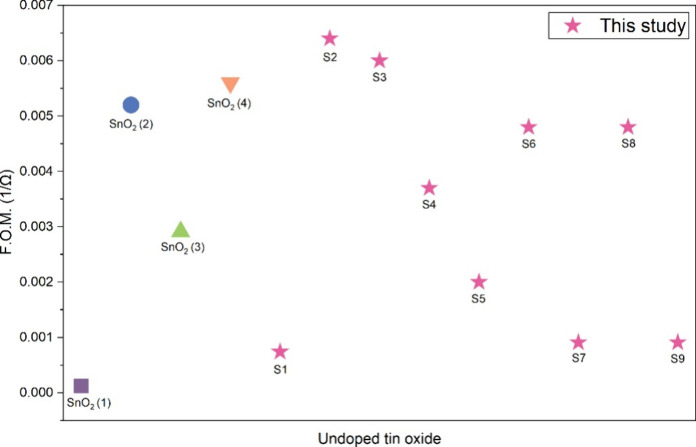
Comparison of the figure of merit (F.O.M.) of SnO_2_ films from this work (**S1**–**S9**) with previously reported undoped SnO_2_ films deposited via AACVD (SnO_2_ (1),^65^ SnO_2_ (2),^64^ SnO_2_ (3),^66^ SnO_2_ (4)^67^).

Overall, the deposition conditions, including solvent composition, deposition temperature, and misting time, strongly influenced the microstructure and chemistry of the SnO_2_ thin films, which in turn influences their functional properties. Films prepared with mixed solvents (e.g., ethyl acetate/MeOH) exhibited lower residual chlorine and hydroxyl content (XPS), leading to larger crystallites (XRD) and smoother, more densely packed morphologies (SEM and AFM). These changes resulted in enhanced mobility and lower resistivity (Hall measurements). Higher deposition temperatures promoted improved precursor decomposition, resulting in larger crystallites, higher carrier concentration, and lower resistivity, but with slightly reduced transparency (UV–vis). Longer misting times increased film thickness and roughness, which adversely affected optical transparency but had a negligible effect on electrical mobility. These relationships were quantitatively supported across all samples (**S1**–**S9**), revealing a clear link between deposition parameters and film performance.

The water contact angles (WCAs) of all of the samples are shown in Figure 11. A clear correlation was found between the surface chemistry (XPS data) and WCAs. The film **S2** with a lower proportion of surface hydroxyl group (23.9%) showed the highest WCA of 104°. However, **S4** with higher hydroxylation (30.19 at %) was superhydrophilic (WCA < 5°). A similar relationship between hydroxylation and wettability has been reported in other thin film systems. Similar observations have been reported previously for SnO_2_ films, where surfaces with lower hydroxylation showed higher WCAs and improved device stability.^68^ No direct correlation was found between the WCA and the content of residual chlorine in the films. Overall, the **S1** and **S9** films with highest Cl content were less hydrophobic than the films with less Cl (such as **S2**, **S3**, **S5**, and **S6**). However, Gong et al. has reported an increase in hydrophobicity upon Cl incorporation and reduced OH content.^69^ This suggests that controlling the hydroxylation is crucial not only for controlling the wettability but also for optimizing the functional (optoelectronic) properties of SnO_2_ films.

**Figure 11:**
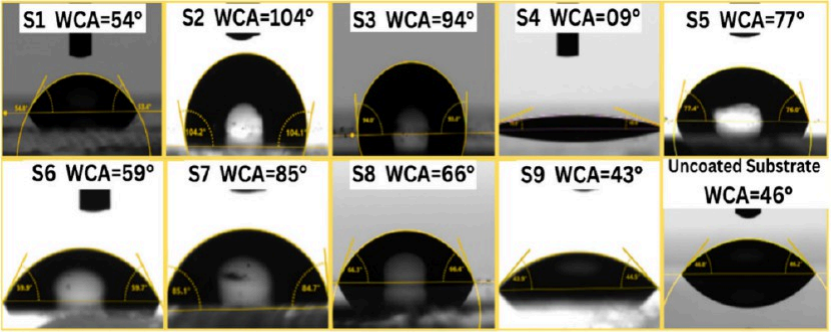
Water contact angle (WCA) images of all the samples and uncoated substrate.

## Conclusions

In this work, SnO_2_ films were deposited by AACVD, and the complex interplay between solvent composition, deposition temperature, and misting time on the structural and optoelectronic properties was studied. The champion films (**S2** and **S3**) achieved minimum resistivities of 9.6 × 10^–4^ and 8.5 × 10^–4^ Ω cm, respectively, with high carrier mobility (22.5 and 23.0 cm^2^ V^–1^ s^–1^) while maintaining excellent optical transmittance of 81.1% and 80.2% at 550 nm. Structural and compositional analyses showed that optimized solvent ratios and deposition conditions promoted dense microstructures and facilitated residual chlorine removal. Lower surface hydroxylation was found to improve the carrier transport properties and hydrophobicity of the films, which can enhance device performance. The results indicate that precise control of solution chemistry and deposition parameters allowed the fabrication of SnO_2_ films exhibiting high transparency and low resistivity, comparable to those achieved in doped systems. These findings demonstrate that appropriate solution chemistry and deposition conditions can facilitate the removal of residual chlorine from the films. The optimized undoped SnO_2_ films, particularly **S2** and **S3**, achieved sheet resistance values close to those of commercial F-doped SnO_2_ and demonstrated strong intrinsic conductivity even without intentional doping. These results highlight the significant potential to further enhance performance through controlled doping strategies, enabling conductivity levels comparable to or potentially exceeding those of commercial FTO. These findings further highlight the effectiveness of AACVD as a versatile technique for depositing high-quality TCO thin films.

## Supplementary Material



## Data Availability

Data supporting the findings of this study are available from the corresponding author upon reasonable request.

## Notes

Preferred orientation analysis, correlation plot between lattice parameters and chlorine content in the films, transmittance and reflectance spectra of uncoated float glass substrate and commercial fluorine-doped tin oxide (FTO) glass, deconvoluted XPS spectra, Kubelka–Munk band gap analysis, and additional experimental details including electrical and optical properties data (PDF)
