# Supplementary material for: Optimized SnO2 Thin Films: Correlating Solution Chemistry and Deposition Conditions with Optoelectronic Properties
Source: ACS Appl Mater Interfaces. 2026 Mar 26;18(13):19364–79. doi: 10.1021/acsami.6c03389 (PMC13067240; doi:10.1021/acsami.6c03389)
Supplement: Supplementary file 1 [file am6c03389_si_001.pdf]

Supporting Information

Optimized SnO_2 Thin Films: Correlating Solution Chemistry and Deposition Conditions with Optoelectronic Properties

Iqra Ramzan[a], *Matthew O. Blunt*[a], *Ivan P. Parkin*[a] *and Claire J. Carmalt*[a]*

[a]Materials Chemistry Centre, Department of Chemistry, University College London, 20 Gordon Street, London WC1H 0AJ, UK.

*Corresponding Author's Email: c.j.carmalt@ucl.ac.uk

In thin films, growth can be anisotropic, meaning that certain crystal planes may grow faster or be more energetically favourable to align in a specific direction. To quantify this preferential orientation, texture coefficient (TC) values were calculated using the **Equation S1**.[1] A TC value greater than 1 for a particular (hkl) plane indicates a strong preferred orientation along that direction.

$$TC_{hkl} = \frac{\dfrac{I_{meas.}(hkl)}{I_O(hkl)}}{\dfrac{1}{N}\sum_{h'k'l'} \dfrac{I_{meas.}(h'k'l')}{I_O(h'k'l')}} \qquad \text{(S1)}$$

Where $I_{meas.}$ is the measured intensity of individual (hkl) plane reflection, I_o is the theoretical intensity obtained from the ICSD data, and N is the total number of reflections included in calculation, *i.e.*, (101), (200), (211), (310), (112), and (301) peaks.

The calculated TC values (Figure 1S) show that films S1-S8 are preferentially oriented along the (200) plane. The (200) plane is among the most favoured, low-surface-energy orientations in SnO_2 and is often associated with the presence of oxygen vacancies and/or dopants. This plane is also reported to have fewer deep trap states at grain-boundary surfaces, which is consistent with higher film conductivity.[2-4]

Deposition temperature was found to have an influence on the preferred orientations. A strong preferred orientation in (200) plane was shown by films deposited at 590˚C (S2) and 550˚C (S8). However, a textural shift at 500 ˚C (S9) was observed (101) plane became dominant and the previously dominant (200) orientation was suppressed. This significant change, along with a notable reduction in crystallite size (15.4 nm), suggests that below 550%˚C, the growth process becomes kinetically controlled. The (101) plane corresponds to a dense crystallographic direction and is widely reported to exhibit reduced surface energy and fewer oxygen vacancies.[5] Agashe *et al.* and Afify *et al.* noted that the (200) plane was particularly sensitive to substrate temperature, which influences the growth of the films.[6, 7]

The preferred crystallite orientation in doped SnO_2 films has been reported to correspond to the (110), (200), (211), and (301) planes, depending on factors such as substrate temperature, dopant concentration, and film thickness.[3, 4] Belanger *et al.* prepared FTO films using CVD and observed that in films with a thickness of around 400 nm, the dominant planes were (200), (211), (301), and (400).[2] Additionally, *Belanger et al.* found that deep trap levels at grain boundaries surfaces were primarily associated with the (110), (211), and (301) orientations, and not with the (200) plane.[2]

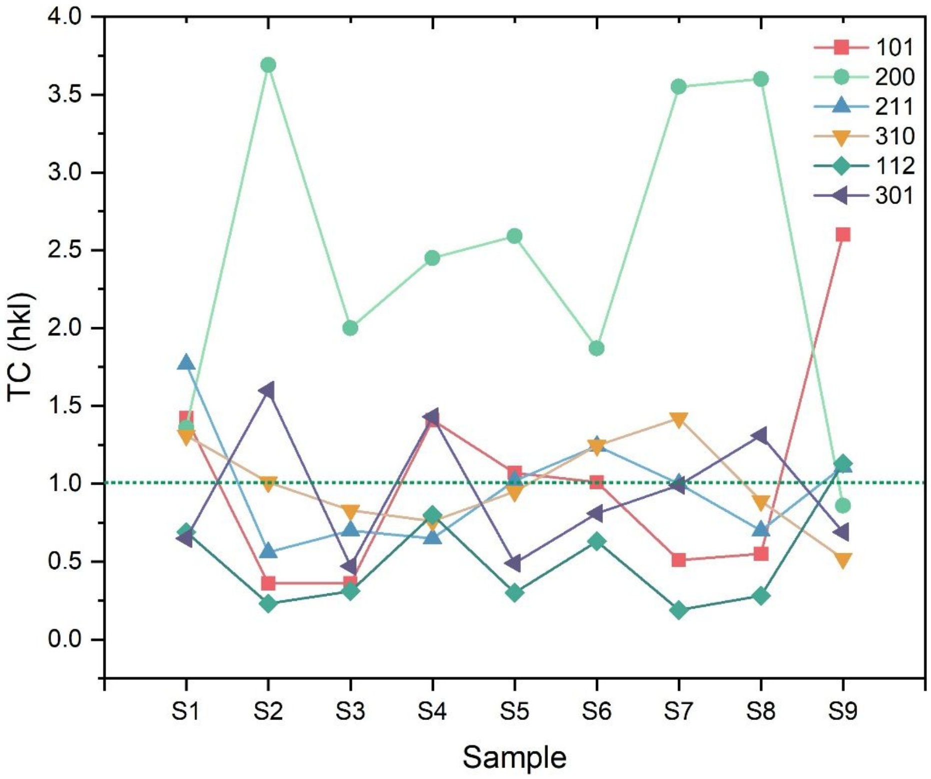

Figure S1. Texture coefficient (TC) values of SnO_2 films for different crystallographic planes (hkl)

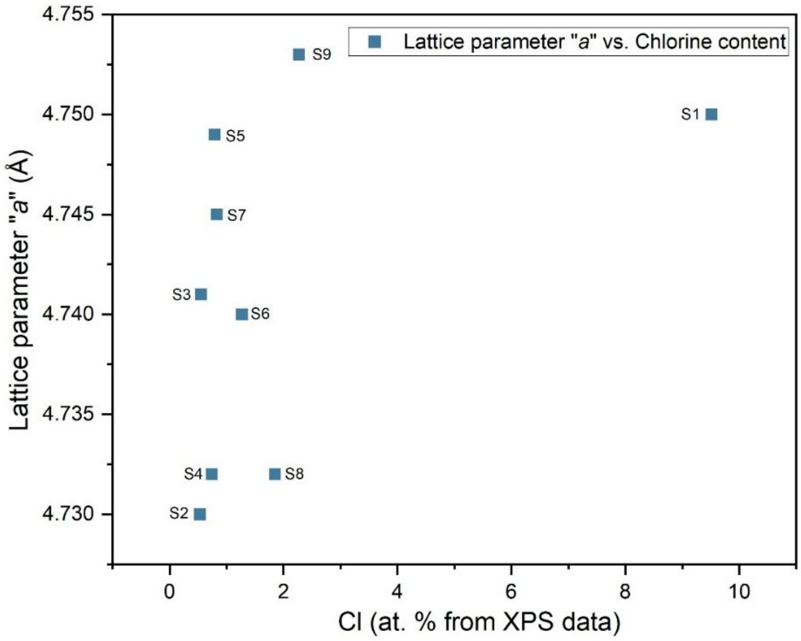

Figure S2. Correlation between lattice parameter *a* and chlorine content (at.% from XPS)

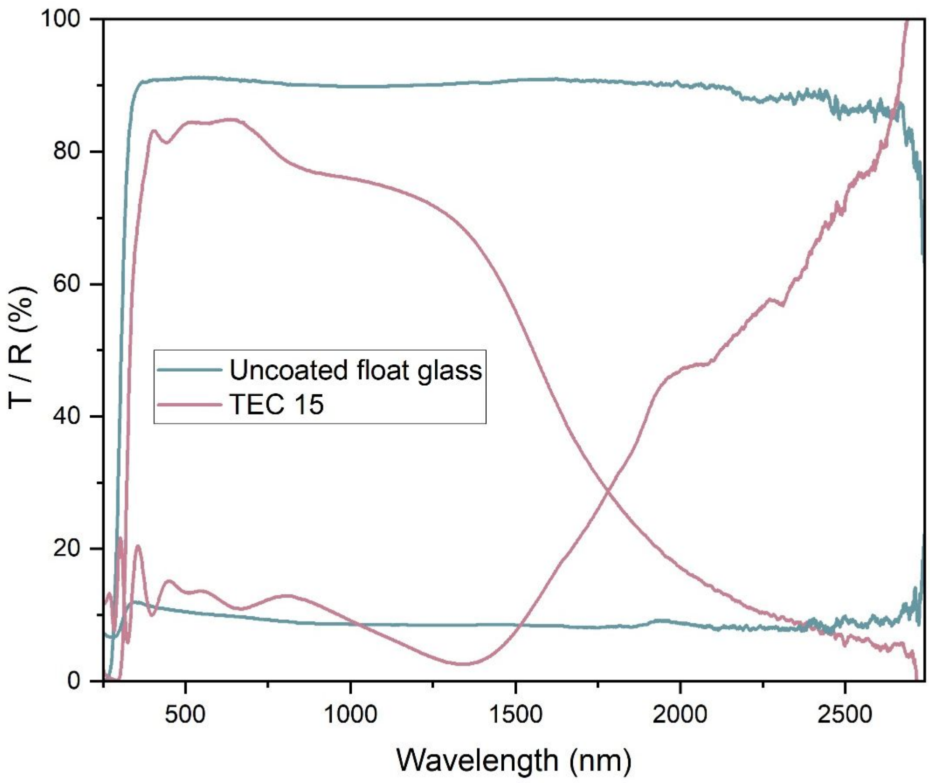

Figure S3. Transmittance and reflectance spectra of uncoated float glass and TEC 15 (from NSG Pilkington)

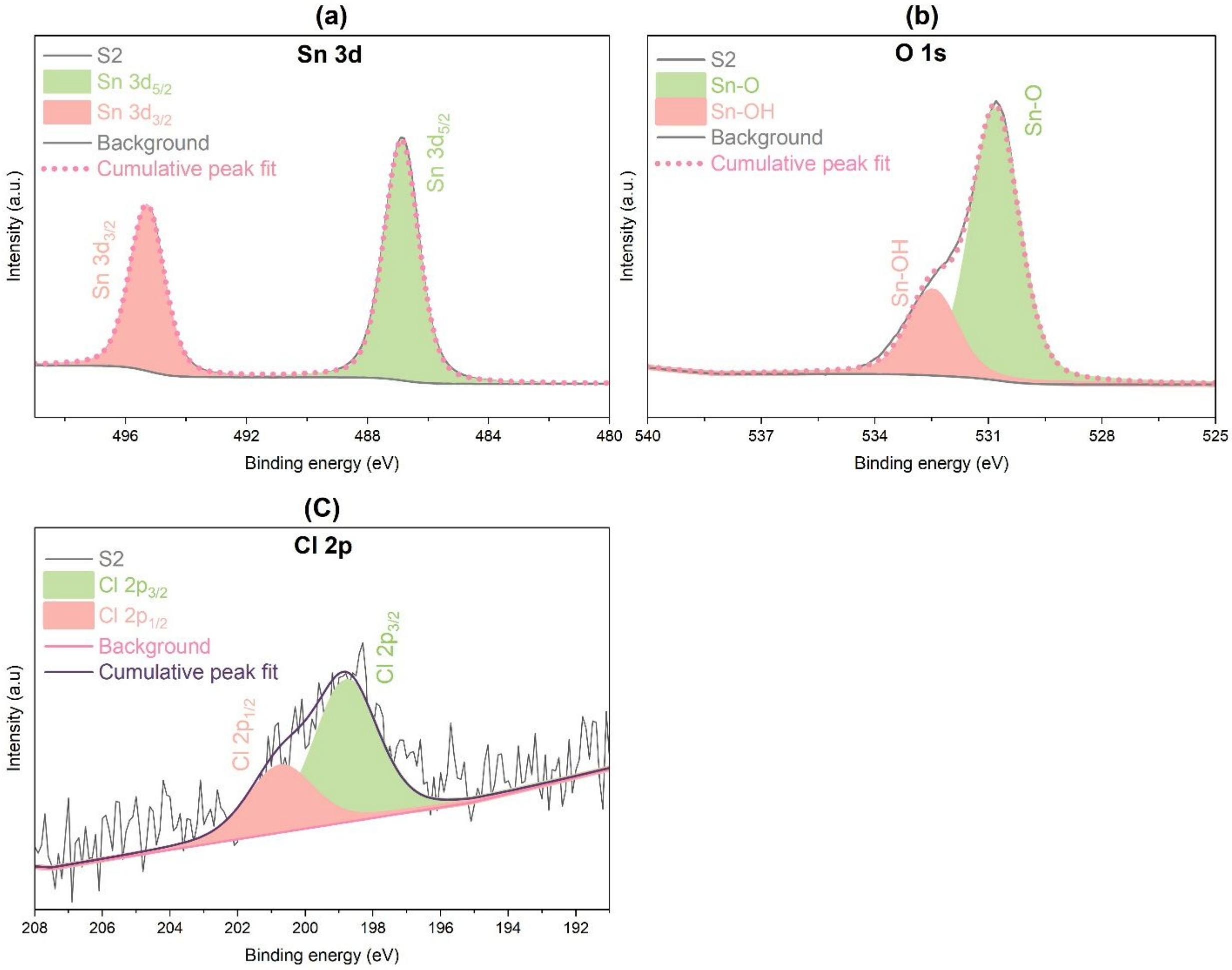

Figure S4. Deconvoluted XPS spectra of (a) Sn 3d (b) O 1s (c) Cl 2p

The band gap values obtained using the Tauc plot method with absorbance data are presented in **Table 2** and the band gaps obtained from Kubelka-Munk (K-M) function (**Equation S2**)[8] with diffuse reflectance data are presented in **Figure S5**.

$$F(R) = \frac{(1-R)^2}{2R} \qquad \text{(S2)}$$

The band gap calculated using the K-M function was slightly lower than the value obtained from absorbance-based Tauc plots, with the difference ranging from 0.12 eV to 0.43 eV. This trend is consistent with typical behaviour observed when using the K-M function, which is sensitive to scattering effects and surface roughness. The largest difference (0.43 eV) was observed for sample **S7**, which can be linked to its higher surface roughness compared to the other films. Interestingly, the difference in band gap values between the two methods is generally smaller for films with less surface roughness (e.g., S1, S2, and S4), with differences of around 0.12 eV to 0.20 eV.

Balakrishnan *et al.*, while studying functional oxides such as V_2O_5, $BaSnO_3$, $PbZr_{0.52}Ti_{0.48}O_3$, $BiMnO_3$, and $BiFeO_3$ using diffuse reflectance spectroscopy, found that the Urbach energy (quantified as disorder-induced absorption near the band edge) ranged from 0.24 eV to 0.48 eV for the studied materials, reflecting the presence of tail states and disorder within the band gap.[9] Similar differences in band gap values, when calculated using different methods, have been widely reported in the literature.[10, 11]

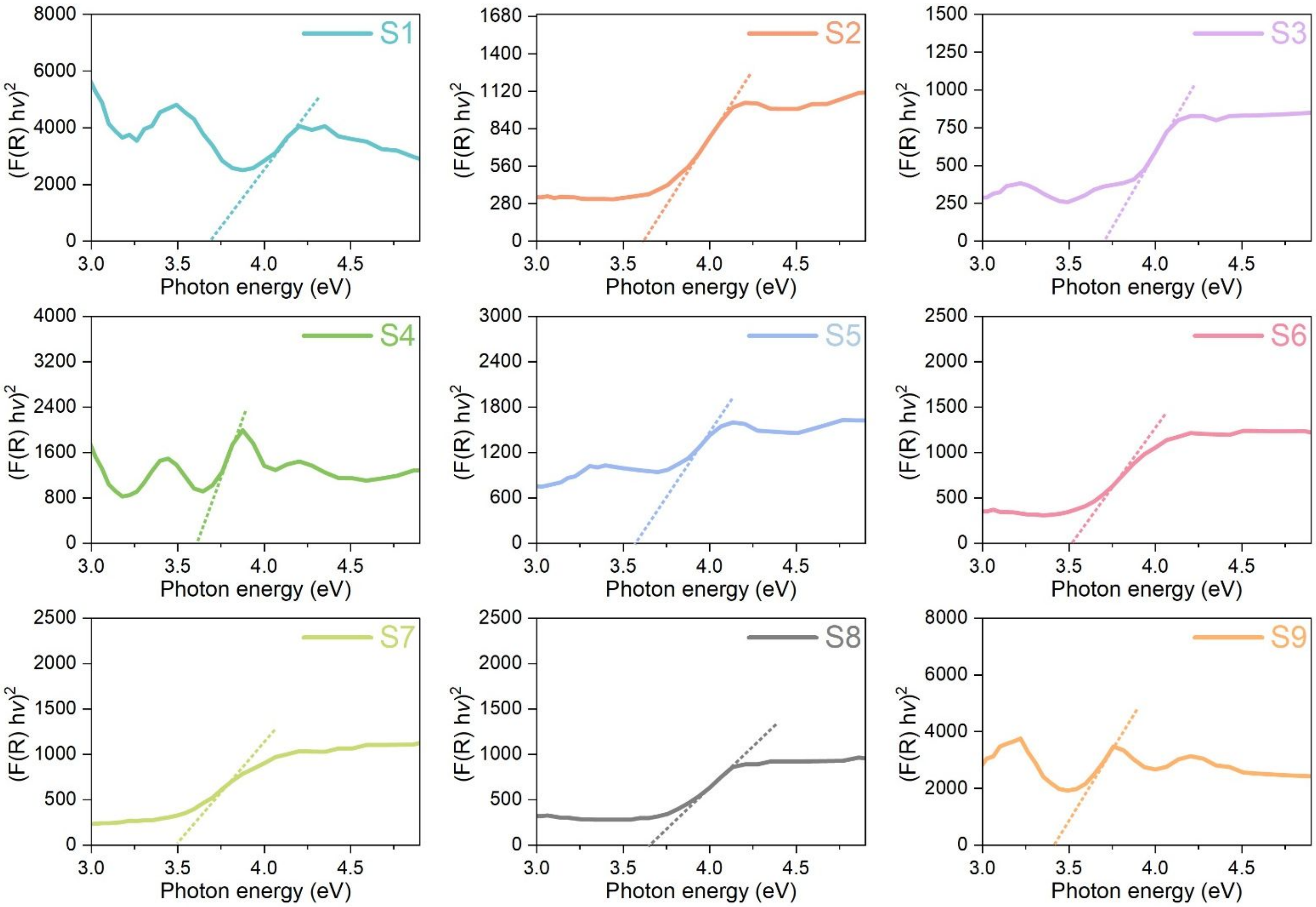

Figure S5. Band gap spectra of **S1-S9** calculated using the Kubelka-Munk function

Table S1. Deposition conditions, film thickness, electrical properties, calculated sheet resistance, and optical transmittance at 550 nm for samples SS1-SS10 (all films were deposited at 590 ˚C).

Film	Solvent composition	$SnCl_4$ (mL)	Misting time (min)	Film Thickness (nm)	Resistivity (Ω cm)	Mobility ($cm^2 V^{-1} s^{-1}$)	Carrier Concentration (cm^{-3})	Sheet Resistance ($\Omega\ \square^{-1}$)	$T_{\lambda550}$ (%)
**SS1**	Ethanol 14mL	0.05	05	400	9.1×10^{-2}	18.0	3.8×10^{18}	2275	82.4
**SS2**	Propanol 14mL	0.05	05	250	5.0×10^{0}	9.2	1.4×10^{15}	2.0×10^{7}	82.0
**SS3**	Dis. Water 14mL	0.05	05	180	$4.3 \times 10^{+4}$	4.45	3.3×10^{13}	2.4×10^{9}	81.3
**SS4**	Toluene 14mL	0.05	05	540	8.2×10^{-3}	20.1	3.8×10^{19}	151.8	58.9
**SS5**	Chloroform 14mL	0.05	05	-	-	-	-	-	89.1
**SS6**	Ethyl acetate 7mL + Methanol 7mL	0.05	03	380	5.9×10^{-3}	20.0	5.3×10^{19}	155.3	79.3
**SS7**	Ethyl acetate 10mL + Methanol 10mL	0.05	15	1400	1.3×10^{-3}	24.5	1.9×10^{20}	9.29	72.9
**SS8**	Ethyl acetate 15 mL + Methanol 15mL	0.05	25	2110	3.1×10^{-3}	27.2	7.4×10^{19}	14.7	67.4
**SS9**	Ethyl acetate 15 mL + Methanol 15mL	0.1	25	2340	5.0×10^{-3}	29.0	4.3×10^{19}	21.4	65.5
**SS10**	Ethyl acetate 15 mL + Methanol 15mL	0.2	25	2550	4.5×10^{-3}	28.0	5.0×10^{19}	17.6	55.8
**Uncoated substrate**	-	-	-	50	2.0×10^{5}	9.4	3.2×10^{12}	4.0×10^{10}	91.1

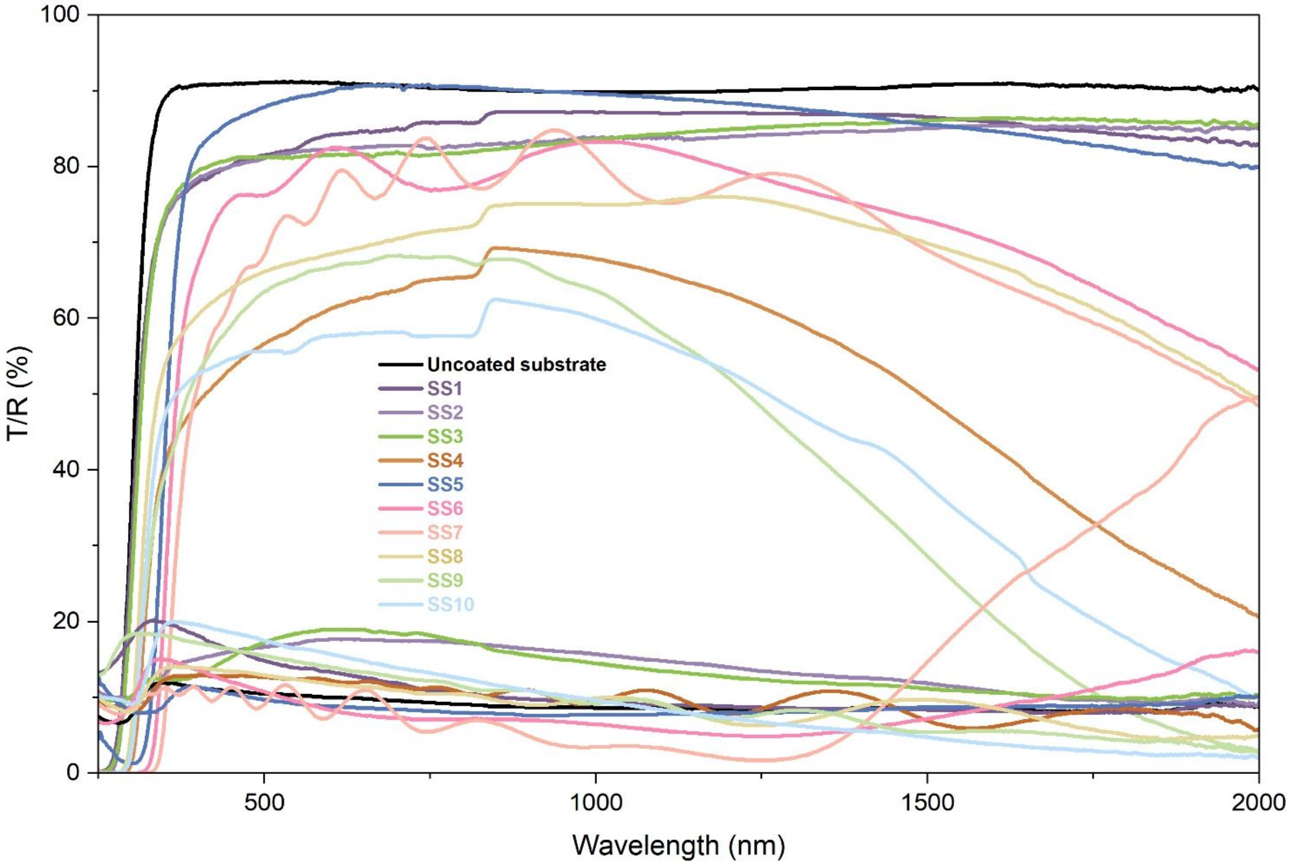

Figure S6. Transmittance and reflectance spectra of **SS1-SS10**

References

(1) Joseph, D. P.; Saravanan, M.; Muthuraaman, B.; Renugambal, P.; Sambasivam, S.; Raja, S. P.; Maruthamuthu, P.; Venkateswaran, C. Spray deposition and characterization of nanostructured li doped nio thin films for application in dye-sensitized solar cells. *Nanotechnology* **2008**, *19* (48), 485707.
(2) Belanger, D.; Dodelet, J.; Lombos, B.; Dickson, J. Thickness dependence of transport properties of doped polycrystalline tin oxide films. *Journal of the Electrochemical Society* **1985**, *132* (6), 1398.
(3) Amanullah, F.; Pratap, K.; Babu, V. H. Compositional analysis and depth profile studies on undoped and doped tin oxide films prepared by spray technique. *Materials Science and Engineering: B* **1998**, *52* (2-3), 93-98.
(4) Bhachu, D. S.; Waugh, M. R.; Zeissler, K.; Branford, W. R.; Parkin, I. P. Textured fluorine-doped tin dioxide films formed by chemical vapour deposition. *Chemistry–A European Journal* **2011**, *17* (41), 11613-11621.
(5) Canestraro, C. D.; Oliveira, M. M.; Valaski, R.; Da Silva, M. V.; David, D. G.; Pepe, I.; Da Silva, A. F.; Roman, L. S.; Persson, C. Strong inter-conduction-band absorption in heavily fluorine doped tin oxide. *Applied Surface Science* **2008**, *255* (5), 1874-1879.
(6) Afify, H.; Terra, F.; Momtaz, R. Substrate temperature effects on the tin oxide films prepared by spray pyrolysis. *Journal of Materials Science: Materials in Electronics* **1996**, *7*, 149-153.
(7) Agashe, C.; Hüpkes, J.; Schöpe, G.; Berginski, M. Physical properties of highly oriented spray-deposited fluorine-doped tin dioxide films as transparent conductor. *Solar energy materials and solar cells* **2009**, *93* (8), 1256-1262.
(8) Zargar, R.; Bhat, M.; Parrey, I.; Arora, M.; Kumar, J.; Hafiz, A. Optical properties of zno/sno2 composite coated film. *Optik* **2016**, *127* (17), 6997-7001.
(9) Balakrishnan, R.; Sahoo, P.; Karuppannan, B.; Dixit, A. Probing electronic transitions and defect-induced urbach tail bands in functional perovskite oxides using diffuse reflectance. *arXiv preprint arXiv:2504.00033* **2025**.
(10) Landi Jr, S.; Segundo, I. R.; Freitas, E.; Vasilevskiy, M.; Carneiro, J.; Tavares, C. J. Use and misuse of the kubelka-munk function to obtain the band gap energy from diffuse reflectance measurements. *Solid state communications* **2022**, *341*, 114573.
(11) Makuła, P.; Pacia, M.; Macyk, W. How to correctly determine the band gap energy of modified semiconductor photocatalysts based on uv–vis spectra. ACS Publications: 2018; Vol. 9, pp 6814-6817.
